# Being an observer of one’s own life—a meta-synthesis on the experience of mechanically ventilated patients in intensive care units

**DOI:** 10.1186/s13054-025-05326-6

**Published:** 2025-03-08

**Authors:** Fritz Sterr, Mareike Hechinger, Lydia Bauernfeind, Christian Rester, Rebecca Palm, Sabine Metzing

**Affiliations:** 1 Faculty of Health, School of Nursing Sciences, Witten/Herdecke University, Alfred-Herrhausen-Straße 50, 58455 Witten, Germany; 2 Faculty of Applied Healthcare Sciences, Deggendorf Institute of Technology, Land-Au 27, 94469 Deggendorf, Germany; 3 Department of Health Services Research, School VI Medicine and Health Sciences, Carl Von Ossietzky Universität Oldenburg, Ammerländer Heerstraße 114-118, 26129 Oldenburg, Germany

**Keywords:** Artificial respiration, Critical care, Intensive care unit, Lived experience, Mechanical ventilation, Meta-synthesis, Review

## Abstract

**Background:**

The experience of patients under mechanical ventilation in the intensive care unit is described as complex and multifaceted, but an overarching and in-depth understanding of the experience is still missing.

**Aim:**

To provide an in-depth analysis and synthesis of patients’ experience when being mechanically ventilated in intensive care units.

**Methods:**

We conducted a meta-synthesis according to the methodological recommendations of Sandelowski and Barroso. Our systematic literature search in Medline, CINAHL, and Cochrane was complemented by hand and citation searches. We included only qualitative studies with a rich description of conscious patients’ experience under mechanical ventilation. Studies on children, step-down units, noninvasive ventilation and non-scientific journal articles were excluded. After the title, abstract and full-text screening by three reviewers, we performed initial, axial and selective coding and in-depth analysis in MAXQDA. The synthesis was supported by multiple discussion rounds.

**Results:**

Of the 2,563 records identified, 20 studies were included in our synthesis. This revealed the central phenomenon of patients being observers of their own lives. They are yearning for a stable picture of reality and developing various situation-specific needs. Finally, patients are finding ways to deal with the situation. These concepts are interwoven in time and are experienced repeatedly in different dimensions.

**Conclusion:**

Patients under mechanical ventilation are highly perceptive. Healthcare professionals are particularly responsible for patients. They should reflect on their role in intensive care and must be sensitized to patients’ differentiated experience.

Registration, Protocol: https://doi.org/10.17605/OSF.IO/G8Q6X

**Supplementary Information:**

The online version contains supplementary material available at 10.1186/s13054-025-05326-6.

## Background

Following international recommendations, sedation regimens for patients under mechanical ventilation (MV) have been adapted in many intensive care units (ICUs) [1–3], and healthcare professionals (HCPs) are reducing or withdrawing sedation of patients as soon as possible [4, 5]. Along with the increasing number of patients under MV worldwide [6–9], this approach leads to a growing number of patients being awake and conscious under MV. This is particularly important, as empirical research indicates that patient's experience has an impact on the duration of MV and ventilator weaning [10, 11].

In detail, patients describe their MV and weaning process as stressful and frustrating [12, 13]. As a consequence, various psychological factors (e.g., patient well-being, stress, the spatial environment, and cognitive ability) are considered relevant by clinicians as they can influence the weaning process [14]. To date, numerous correlations have been investigated. Cognitive alterations (e.g., delirium) influence the duration of ventilator weaning [15, 16]. In addition, psychological distress, anxiety, depression and hallucinations are negatively associated with the outcome of spontaneous breathing trials [17] and overall weaning success [18]. It is therefore crucial to know the individual patients and understand their experience in a differentiated way [19].

Meanwhile, a number of empirical findings on the experience of ventilated ICU patients are available. In addition, three meta-syntheses [20–22] have been published that analyze these studies in more detail and provide further insights. However, the findings of the identified meta-syntheses are limited due to expanded patient perspectives. Although they identify essential aspects of the experience, these articles remain superficial in their analysis and description. There is no in-depth understanding of patients’ lived experience, and the relationships between core components and sub-dimensions have not yet been sufficiently investigated. In addition, restrictions were placed on the countries (e.g., only Scandinavian studies [20]) or years of publication (e.g., only studies from 2013 to 2019 [21]), which further limits these studies.

## Aim and research question

Since an in-depth analysis of ICU patients’ lived experience under MV is still lacking, we aim to identify, summarize and synthesize qualitative studies on the experience of mechanically ventilated patients. In this regard, we address the following research question: “What do adult intensive care patients experience when being conscious under mechanical ventilation?”.

## Design and methods

### Synthesis methodology

We conducted a systematic review and meta-synthesis according to the PRISMA (Preferred Reporting Items for Systematic reviews and Meta-Analyses) [23] and ENTREQ (Enhancing transparency in reporting the synthesis of qualitative research) [24] guidelines. In this process, we followed the methodological guidelines of Sandelowski and Barroso [25], and the ‘lessons learned’ by Herber and Barroso [26]. In detail, we applied the recommended six steps to conduct our meta-synthesis [25]. After (1) formulating a purpose, we (2) systematically searched for relevant qualitative studies. We then (3) screened the identified studies and (4) classified their findings. Finally, we (5) conducted a meta-summary and (6) developed our meta-synthesis.

### Registration and protocol

Following the current PRISMA recommendations [23], we developed a research protocol and registered our meta-synthesis in the Open Science Framework in April 2024. (10.17605/OSF.IO/G8Q6X).

### Eligibility criteria

To develop our research question and define the eligibility criteria, we followed the PICo scheme (Population, Phenomenon of Interest, Context) as recommended for qualitative research [27, 28].

In our review, we included studies on adult patients who were mechanically ventilated by an endotracheal tube or tracheostoma. Studies on children and patients undergoing noninvasive ventilation were excluded.

Patients’ experience under MV was defined as the phenomenon of interest. For this purpose, we considered not only studies that examined the experience of MV itself, but also studies that investigated the experience during MV treatment. In addition, the experience must be reported by the patients themselves. We excluded studies that did not report any experiences or that reported the experiences of patients who were not invasively ventilated. We also excluded studies in which patients did not recall their MV experience within 6 months of ventilator weaning, as patients’ memories and recollections change over time (recall bias) [29, 30].

Our context of interest was the entire treatment process during the ICU stay in an acute care hospital, as long as the patients were mechanically ventilated. Subsequently, we excluded research conducted on patients undergoing MV in step-down units, intermediate care units, outpatient care, or long-term care facilities.

Furthermore, only original qualitative studies in English, German or Spanish were included in our meta-synthesis. These had to provide a rich description of their results [31, 32] in order to perform the intended in-depth analysis. Reviews, gray literature, and nonscientific journal articles were excluded. Finally, studies that did not report transparently on their methodological approach or ethical considerations were also not considered for inclusion.

### Approach to searching and search strategy

To identify potentially relevant studies, we developed a comprehensive and systematic search strategy. Therefore, two reviewers (FS, LB) conducted formative searches in Medline (via PubMed) and the Cumulative Index to Nursing and Allied Health Sciences (CINAHL, via EBSCO host) to identify relevant keywords and terms. Afterwards, we developed a three-armed systematic search for each intended database within the research team. We also included the terms proposed by Sandelowski and Barroso to search for qualitative research [25]. According to the identified research gap, we did not use any filters or restrictions in our search. The final search strings and their results are illustrated in Additional file 1.

### Data sources

We conducted a systematic literature search within the three databases Medline (via PubMed), CINAHL (via EBSCO host), and Cochrane Library on January 23rd, 2024. To ensure a sensitive research strategy [33, 34], we deliberately refrained from using any filters in the databases (e.g., publication type, timeframe). After title and abstract screening, we conducted an additional hand search [35] in LIVIVO and Google Scholar, as well as a citation search [36] with identified reviews and included studies in May 2024. The search strategies are illustrated in Additional file 1.

### Study screening methods

After records from our systematic search were exported and screened for duplicates, two reviewers (FS, LB) independently conducted title, abstract and full-text screening in the Rayyan web application. When the two reviewers disagreed, a third reviewer was consulted to resolve conflicts (MH).

### Quality appraisal

As recommended by Sandelowski and Barroso [25], we assessed the quality of each study. Therefore, we used the Critical Appraisal Skills Programme (CASP) and applied its checklist for qualitative studies to each included study [37, 38]. Two reviewers (FS, MH) independently appraised each article. Disagreements were again discussed and resolved with a third reviewer (LB). In the assessment, four items were identified as not fulfilled or unclear in one study. All other studies were of high quality. The final appraisal reports are provided in Additional file 2.

### Data extraction

To provide information on the individual study characteristics, we extracted data on the authors, publication year, country, aim of the study, design, population, and data collection and evaluation methods (see protocol, 10.17605/OSF.IO/G8Q6X). For the in-depth analysis and synthesis, we examined the statements of findings of the included studies.

### Coding, classifying the findings, study comparison and derivation of concepts

The study results were analyzed in MAXQDA (version 24). After data import, the analysis and synthesis were performed by three reviewers (FS, MH, LB) via an inductive approach [25]. The coding procedures were based on those of Saldaña [39]. As the first cycle method, initial coding was conducted in the results chapters of the included studies. We analyzed the statements of findings and the integrated patient quotes line by line and sentence by sentence with the aim of going beyond what is said and discovering deeper and hidden patterns. While axial coding was applied as the second cycle method, constant comparison was used to identify similarities and differences as well as to identify categories and subcategories. As the third cycle method, selective coding promoted a deeper theoretical level of abstraction that developed more general categories and concepts. In addition, we extracted some of the patients’ statements directly from the results chapters to illustrate our findings.

To ensure quality and transparency, each study was analyzed by at least two reviewers. A first reviewer carried out the coding and wrote memos using the growing code tree. The second reviewer confirmed, complemented or rejected the codings and memos. A third reviewer was consulted if discrepancies arose. Multiple discussion rounds were held to analyze and synthesize the findings.

## Results

### Study selection results

**Fig. 1:**
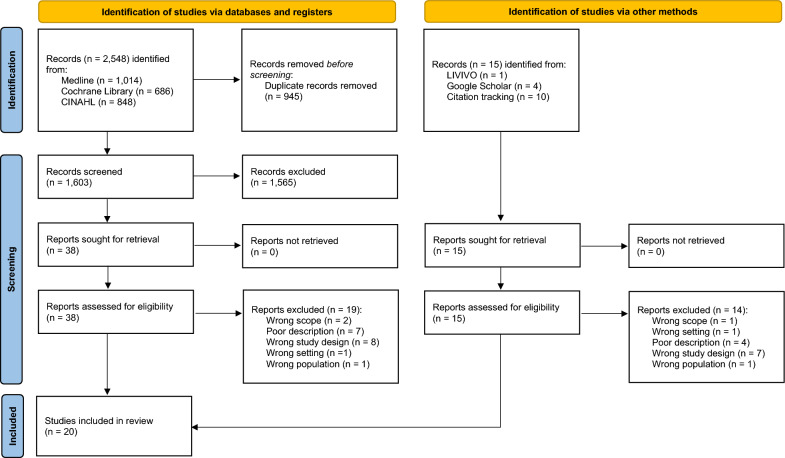
PRISMA flow diagram of the search and screening process (Page et al., [20])

The systematic literature search yielded 2,548 records, and the additional hand search and backward citation tracking revealed a further 15 records. Of the overall 2,563 titles, 945 duplicates were removed and 1,618 titles and abstracts were screened. Of the 53 studies included in the full-text screening, 33 studies were excluded (see Additional file 3). Finally, a total of 20 studies were included in our meta-synthesis [40–59]. The entire search and screening process is shown in Fig. 1.

### Study characteristics

The included studies were published between 2006 [41, 43, 51] and 2023 [56], and comprise a total of 275 examined patients. Half of the studies were conducted in Scandinavia, six in Denmark [49, 50, 54–57] and four in Sweden [44, 52, 53, 58]. While interviews were carried out in all 20 studies, four studies conducted additional field observations [47, 49, 55, 56]. In the studies without field observations, the interviews were unstructured in six cases [41, 43, 51–53, 59], semi-structured in nine cases [40, 42, 44, 45, 48, 50, 54, 57, 58] and structured once [46] (see Table 1).

**Table 1:** Characteristics of included studies

Author, year, country	Aim	Study design	Population	Data collection	Data analysis
Aslani, 2017, Iran [40]	To investigate the psychological experiences of patients following open heart surgery as they are under ventilation	Phenomenological approach	15 patients	In-depth, open and semi-structured interviews after full recovery in the surgical ward	Critical hermeneutical analysis (Diekelmann)
Ballard, 2006, USA [41]	To understand patients' recollections and to determine whether they experienced pain, anxiety, or negative or positive recollections of care despite the administration of sedatives and/or analgesics	Phenomenological approach	11 patients	Unstructured open-ended interview in the critical care unit or another unit	Constant comparison method; cross-case intuitive analysis and individual case analysis in alternation
Dale, 2020, Canada [42]	To examine the procedural oral pain recollections of intubated and mechanically ventialted patients	Qualitative descriptive design	33 patients	Combination of semi-structured interviews with object elicitation to trigger memories and tacit insights within 7 days of ICU discharge	Content analysis
Donnelly, 2006, Australia [43]	To investigate the lived experience of patients undergoing a tracheostomy tube change	Phenomenological approach	4 patients	Unstructured interviews when convenient to patients during hospital stay	Hermeneutic analysis (Ricoeur)
Engström, 2013, Sweden [44]	To describe the reported ICU experiences of patients undergoing mechanical ventilation	Qualitative descriptive design	8 patients	Semi-structured interviews 6 months after MV cessation	Content analysis (Downe-Wamboldt)
Gilder, 2021, New Zealand [45]	To describe the patient experience of the endotracheal tube and suction, following mechanical ventilation in post-operative cardiac surgical patients	Qualitative descriptive design	10 patients	Semi-structured interviews 4 to 6 days postoperatively	Inductive thematic analysis
Guttormson, 2015, USA [46]	To describe the patient experience of communication during mechanical ventilation	Secondary data analysis of a mixed-methods descriptive study	31 patients	Structured interviews along the Intensive Care Experience Questionnaire (ICEQ) after transfer from ICU, follow-up questions during instrument completion, open-ended questions	Modification of qualitative content analysis
Hajiabadi, 2017, Iran [47]	To explain the experiences of conscious patients undergoing mechanical ventilation in ICUs regarding their visits with their family members	Qualitative design	15 patients	Observations, and 15 in-depth, semi-structured interviews in the ICU and normal wards, and 4 complementary interviews at patients' homes	Qualitative content analysis
Hajiabadi, 2018, Iran [48]	To explain the experiences of mechanically ventilated patients in ICU	Qualitative design	15 patients	15 in-depth, semi-structured interviews in the ICU and normal wards, and 4 complementary interviews at patients' homes	Qualitative content analysis
Holm, 2017, Denmark [49]	To explore non-sedated mechanically ventilated patients' communication with nurses in the ICU	Phenomenological hermeneutic approach	9 patients, 13 nurses	9 semi-structured interviews with patients 1–2 days after extubation, 5 semi-structured focus group interviews with nurses, 12 field observations	Ricoeur's theory of interpretation
Holm, 2015, Denmark [50]	To explore adult intensive care unit patients' experience of being conscious during endotracheal intubation and mechanical ventilation	Phenomenological hermeneutic approach	4 patients	Semi-structured interviews 1–2 days after extubation	Ricoeur's theory of interpretation
Johnson, 2006, Australia [51]	To explore the meanings former patients attributed to being on long-term MV in a critical care unit	Phenomenological approach	9 patients	14 unstructured in-depth interviews (9 initial, 5 follow-up) 2 weeks to 2 months after hospital discharge	Thematic analysis (van Manen)
Karlsson, Bergbom, Forsberg, 2012, Sweden [52]	To illuminate the lived experience of patients who were conscious during MV in an ICU	Phenomenological hermeneutic approach	12 patients	Unstructured interviews approximately one week after ICU discharge	Phenomenological hermeneutic analysis (Ricoeur)
Karlsson, Lindahl, Bergbom, 2012, Sweden [53]	To describe patients' statements about their situation while conscious and receiving MV	Hermeneutic approach	14 patients	Video-recorded, unstructured interviews between 2 and 14 days after arrival in the ICU	Qualitative and quantitative content analysis; hermeneutic approach to analyze facial expressions
Kjeldsen, 2017, Denmark [54]	To investigate patients' experience of thirst while being conscious and mechanically ventilated	Hermeneutic approach	12 patients	Semi-structured open-ended interviews in ICUs after extubation	Hermeneutical analysis using manifest and latent content analysis
Laerkner, 2017, Denmark [55]	To explore patients' experiences of being awake during critical illness and mechanical ventilation in the ICU	Interpretative descriptive study	20 patients	20 initial and structured interviews one week after ICU discharge; 13 semi-structured follow-up interviews two to four months after ICU discharge; 28 participant observations	Qualitative, descriptive and interpretive thematic analysis
Lehmkuhl, 2023, Denmark [56]	To gain in-depth understanding of the phenomenon of mobilisation when conscious and mechanically ventilated patients are mobilised in ICU	Phenomenological hermeneutic approach	12 patients	200 h of participant observations with 12 patients, 2 semi-structured interviews with each patient 2–5 days after ICU discharge and 1–2.5 months after hospital discharge	Interpretative approach (Ricoeur)
Schou, 2008, Denmark [57]	To provide a contemporary description of the patient experience of mechanical ventilator weaning	Qualitative descriptive design	10 patients	Semi-structured in-depth interviews 2 to 5 months after hospital discharge	Hermeneutic interpretive phenomenological approach
Tingsvik, 2018, Sweden [58]	To explore the meaning of being a patient on mechanical ventilation during the weaning process in the ICU	Phenomenological hermeneutic approach	20 patients	Semi-structured interviews 2 to 4 months after ICU discharge	Inductive analysis according to hermeneutic phenomenology (van Manen)
Wang, 2009, China [59]	To examine patients' subjective experience of MV	Phenomenological approach	11 patients	Unstructured in-depth interviews 3 to 14 days after ICU discharge	Phenomenological analysis (Giorgi)

### Meta-summary and derivation of concepts

In our analysis of the included studies, we coded 3,058 text passages and patient quotes that are reported in the study findings. These were then assigned to 18 subcodes, three major concepts and one central phenomenon. Table 2 contains an exemplary and reduced code tree, which illustrates the path from a patient statement or a study finding via the coding to the subcodes and the major concept.

**Table 2:** Example of the coding process and tree

Major concept	Sub-codes	Coding	Finding or quote
Yearning for a stable picture of reality	Becoming aware of one’s own situation	Remembering fragments	Another likened the experience as remembered fleeting moments, “It’s just, it’s just passing moments you remember, you know, fleeting moments, you know.” [41]
		Becoming aware of one’s transition from unconsciousness to consciousness	Participants described many situations in which they felt as if they were moving ‘in and out of consciousness’. [51]
	Differentiating one’s own emotions	Being afraid of missed care	“I had a fear that things did not go well. The personnel, who were really busy, were likely to forget something.” [47]
		Feeling stressed when not being able to speak	Communication or least the ability to speak is a fundamental need for most people; for Brian the stress of not being able to speak “drove me nuts.” [43]
	Perceiving various bodily sensation	Perceiving the tube as narrowing	“Well you can feel that there is a lump down there. It is as if it is too big. But I knew what it was.” [50]
		Feeling uncomfortable due to thirst	“I was thirsty. I asked for a little water. […] They gave me two drops just to wet my tongue and that was all. It was like showing water to a thirsty person and then keeping the water out of reach.” [48]
	Trying to make sense	Findings explanations	Discomforting oral health changes were perceived to result from dehydrating stressors such as a continuously open mouth and injury from orally placed devices. [42]
		Not being able to process information	They heard alarms and signals from medical equipment but had difficulty understanding what they meant. [58]

For each subcode, codings were assigned in at least half of the included studies, and for 13 of the 18 subcodes, codings were evident in 75% or more of the studies. The detailed meta-summary can be found in Additional file 4.

### Meta-synthesis of conscious patients’ experience under MV

In our analysis and synthesis of the data, we identified one central phenomenon: patients are observers of their own lives. This is further differentiated and explained in more detail by the three concepts ‘yearning for a stable picture of reality’, ‘developing various situation-specific needs’ and ‘finding ways to deal with the situation’.

**Fig. 2:**
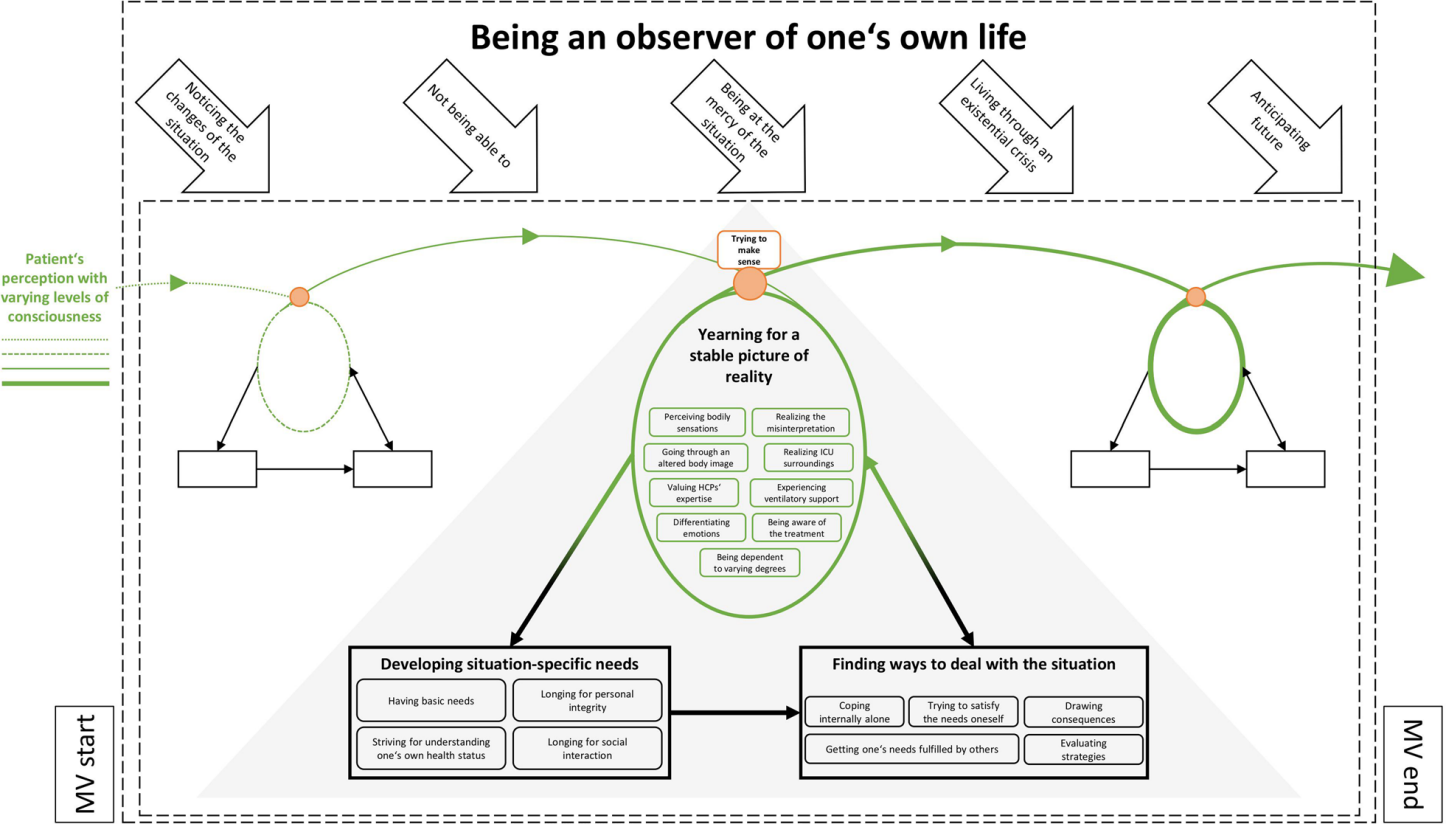
Conceptual model of patients’ experience under mechanical ventilation. The green line represents patients’ perception. This is dependent on their consciousness throughout mechanical ventilation and changes over time. It can be fragmented and superficial (thin, dotted line) or continuous and very clear (bold, continuous line)

Figure 2 illustrates the interconnection of these concepts. It depicts the observation of the patient's own life throughout the MV process. Over the entire period, patients’ perceptions depend on their dynamic state of consciousness. They are developing their picture of reality in recurring loops, thereby constantly integrating the perceived aspects (e.g. ICU surroundings). From this picture, patients can derive specific needs to which they respond with different strategies. However, they can also proceed directly into the strategies without developing needs in between. In both cases, the experiences from the needs and the dealing strategies are integrated into one's own picture of reality. This triangle forms the core of the repetitive loops, but varies in its intensity and extent. All steps are continuously observed by the patients and run through various stages. While patients perceive their changed situation at the beginning, the situation becomes increasingly critical and they make assumptions about their own future.

## Being an observer of one’s own life

Overall, the included studies revealed that patients under MV are in a continuous observer role because of their impaired ability to actively contribute to their situation. Patients' perception is complex; they (try to) differentiate between their own body and psyche as well as their situation, the environment, and the people in it. In addition, they usually evaluate their experiences and observations. Finally, they derive predictions about their own future from what they know and have experienced, although these predictions may differ from those of HCPs.

### Noticing the changes of one’s situation

Patients are aware of their own situation. They are thrown out of their previous lives [47, 48, 52, 55, 58, 59] and confronted with large and small changes [43, 45, 55, 58]. They perceive their situation as variable and dynamic, especially if it becomes worse [40, 44, 47, 54, 58, 59]. “You felt a bit like… well, like a vegetable, not being able to manage yourself… and of course at home I’m… there I look after myself and manage everything… I wanted to go home again!” [52]

### Not being able to

An important element of the care process is the evaluation of one’s own physical and mental abilities. Patients become aware of the limits of their own abilities and realize what they can no longer do. It is a continuous understanding of actions that they cannot (or no longer) perform [40–46, 48, 49, 51–59]. “I was drugged up. I couldn't… like I was in a paralyzed state. I couldn't do anything” [41].

### Being at the mercy of the situation

Owing to their own inabilities, treatment, staff and environmental conditions, patients are at the mercy of the situation. They feel powerless [42, 58, 59], give their fate out of their hands [41, 44, 51, 52] and have to accept that they have hardly any influence or options left [41, 43, 47, 51, 52, 59]. “I felt like I could see me and I was dying and there was nothing I could do” [41].

### Living through an existential crisis

Due to the perceived changes, their own inabilities and the fact that they are at the mercy of others and the situation, the patients under MV are living through an existential crisis. They are in urgent need of help [46, 52] and want to escape the situation [44, 48, 50, 56, 58, 59]. Death is omnipresent, patients think a lot about illness [44, 48, 49, 51, 59] and know little or nothing about their own chances of survival [41, 49, 51, 52]. They are having the impression of moving between life and death [40, 41, 47, 48, 50–53, 55, 58, 59], thereby asking questions about the meaning of life [43, 59]. “I felt like I was going to die. I felt myself very close to death and I felt I was dying” [48].

### Anticipating future

As a result, patients are anticipating their own future. Most patients are gradually developing negative prospects [40, 45, 48, 51, 58] and are afraid of lasting impairments [48]. They are considering that MV could be forever [40, 51, 52, 58] and that they will not be able to leave the ICU once again [40]. However, they also feel hope when their health status improves and their previous life becomes more attainable [40]. Patients look forward to the removal of their cannulas [43, 49, 50, 53] and are confident when knowing the outcome of the treatment [40]. “To be honest, I gradually got disappointed. I wondered maybe I would be breathing with this device until the end of my life, maybe I would not be able to get rid of here once again” [40].

## Yearning for a stable picture

Patients under MV find themselves in highly dynamic and unfamiliar situations. The first key concept of the central phenomenon comprises patients’ attempts and desires to grasp and understand their new reality. As they become increasingly aware of their own situation, patients differentiate between a variety of emotions and physical sensations. From this complex self-observation, patients begin to derive explanations, try to find meaning and develop a coherent and stable picture of their altered reality.

### Becoming aware of one’s own situation

The conscious experience of patients under MV begins when they are becoming increasingly awake and attentive to their own situation. To become fully aware of their own situation, patients must reconsider what they have experienced and recognize their own misperceptions as such. They also need to take a differentiated view of themselves and their surroundings and integrate external information.

#### Realizing the misinterpretation of the situation

Realizing the misinterpretation of the situation is based on a changed situation and one's own interpretation as well as the realization that one’s initial interpretation was wrong [40–42, 44–46, 48–55, 57–59]. This is made more difficult by the continuous transition between being more or less conscious [40, 41, 45, 47, 48, 51, 58]. Gilder [45] describes this state as “emerging from the fog”, whereas Ballard [41] calls it “back and forth between reality and the unreal”. It is challenging for patients under MV to precisely define reality [48, 51, 54, 57–59], because they can only interpret what they hear, see, smell, and generally perceive and how they relate it to what is in their memory [40, 59]. Aslani [40] cites an interviewee misinterpreting communication: “They said that old man was very critically ill and there was no hope for his survival and we did not think that he would be detached from the device once again. I thought they were talking about me, my heart palpations increased.”

#### Being aware of one’s own situation

Once patients realize their own misinterpretation, they become increasingly aware of their own situation. This reveals a complex perception that can be subdivided into six components. First, patients are realizing the ICU surroundings; they perceive their immediate environment, the bed, technology and devices, noises, routines in the unit and the people in it [40–53, 55, 57–59]. Second, patients also experience the ventilator support. They differentiate between the endotracheal tube and the respirator, seeing it as a foreign, disruptive object or as valuable support. Patients remember their MV to varying degrees and differentiate between ventilation modes and times [40–46, 48, 50–59].

Third, patients are going through an altered body image. They are clearly limited in their strength and experience their body as strange, tired and weak. Patients no longer have a voice and are decoupled as a person; they simply are and have no task or goal [40, 41, 43–45, 47–53, 55–59]. Fourth, patients are being dependent to varying degrees. They are subject to various physical restrictions and thus lose control over their lives, money and time. Patients perceive this control by others as a significant restriction, but it can also provide them with security. Once they are given back their autonomy, the feeling of dependency is reduced [40–59].

Fifth, patients are aware of their treatment. They notice the application of medication or oral care and pay attention to the little things at the same time. Patients become familiar with the workflow of HCPs and recognize their different roles. They also interpret the function of the technical devices and are aware of continuous monitoring [40–52, 55–59]. Sixth, patients are constantly valuing HCPs’ expertise. They have different expectations regarding HCPs and their competencies. Patients perceive well-trained and competent HCPs, but also experience disrespectful, rude and inattentive HCPs. They differentiate between nurses and physicians and recognize differences from HCPs in general wards [42, 43, 47, 49, 52, 53, 55, 57–59]. Above all, patients’ experience is not always congruent; it is dynamic and changes over the course of treatment.

#### Being aware of one’s own improvements

Patients progress during their treatment by leaving their old health condition behind, accepting the changes and recognizing the improvements as such [45, 51–53, 56, 58, 59]. These are not always linear and constant, but they are important for patients. They describe their own recovery process in a differentiated way and are aware of the efforts involved [44, 45, 48, 56]. They draw hope and motivation from an increasingly stable state of health [40, 45, 47, 53, 59] but are also tired from training and need time to recover [44, 45, 48, 56, 58]. "I lifted my legs up and brought them down. Then, I was tired because of them. The exercises for my legs were very exhausting for me. I could not do it at all" [48].

### Differentiating one’s own emotions

Patients also experience a variety of feelings and emotions. On the one hand, these feelings can be very indifferent, as patients are overwhelmed by the many impressions around them. On the other hand, they can also name and differentiate their feelings very clearly if they manage to organize these impressions. Specifically, patients experience anxiety [40–42, 44–48, 51, 52, 54, 56–59], frustration [41, 48–52, 54, 59], stress [41–43, 46, 51] or loneliness [40, 41, 47, 49, 54, 57–59]. They feel vulnerable [41, 42, 44, 47, 51, 55, 58, 59], desperate [40, 47] or full of panic [42, 44, 51, 52, 54, 58]. However, they also feel happy [44, 45, 47], hopeful [40, 43, 45, 47, 52, 53, 56, 58, 59], grateful [41, 44, 46, 52–54] or secure [44, 46, 47, 52]. These feelings are dynamic, sometimes stronger and sometimes weaker, suddenly appearing or disappearing completely. Patients can also recognize the cause of individual feelings and assign them to situations. A patient reported on his nightly fear of sleeping and explained the reason for it: “It’s so vivid. I mean these dreams I had that time were so bad. They were really horrific and I will never forget it because I wasn’t asleep” [51]. In addition, they often feel much stronger than they are used to, and in some cases, they experience previously unknown feelings, particularly because they are in a dependent, life-threatening situation. “It is because you feel humiliated. You lie there, and they ask questions about all sorts of things, but you have already tried to express that it isn’t what it is about… And you think: When do you get it!?” [49]

### Perceiving various bodily sensations

Similar to their emotional awareness, patients perceive a variety of physical sensations and are able to consciously differentiate between them. The intensity of the perception varies depending on the medication. However, the origin of the sensation and its localization are determined fairly accurately. Specifically, patients feel warmth or cold in their environment [48], and they experience pain due to interventions or their position [40, 42–46, 48–59]. Patients sense their gastrointestinal tract [48, 50–53, 56, 57] and know when they need to go to the toilet [48]. They feel that their lungs expand and are ventilated [42, 48, 53, 58]. Patients are also aware of changes caused by medication; they feel sleepy, weak, tired or suppressed [41, 42, 45–48, 50]. “Well, no, I was not quite under enough. Not to not know anything. I was under it just enough that I knew some things. Well, I guess I remember them going in and out of my mouth, changing the tubes” [41].

### Trying to make sense

The patients try to derive explanations from their self-perception under MV and not only experience their situation but also organize and understand it. In this process, they are dependent on information they receive from others [40, 43, 44, 46, 51, 52, 58] and their ability to understand this information [41, 44, 46, 51, 58]. If patients lack information, they refer back to their own knowledge and form hypotheses about what has happened [40–45, 48, 52, 54, 58, 59]. In addition, they orient themselves to their surroundings [46] and to time, and try to assign their feelings and sensations to situations [41, 59]. One patient stated: “When I woke up, I did not know where I was. First, I thought I slept on the furniture of our home. Gradually, I realized that here is somewhere else. I could not move at all. I thought I had a myocardial infarction because my tongue got numb and I could not talk. I realized that a tube had been put in my mouth” [40].

## Developing various situation-specific needs

As the second key concept, the development of the needs of patients forms an essential part of their experiences under MV. The needs of patients result from their self-perception and form the basis for their subjective evaluation of treatment in the intensive care unit. In detail, patients develop a variety of physical, psychological and social needs. These can be one-off, recurring or ongoing, with varying degrees of urgency and intensity. In addition to basic needs, patients describe a desire for information and understanding. They also strive for personal integrity and value social interaction.

### Having basic needs

Throughout their treatment in the ICU, ventilated patients have basic needs. They need help with personal care [44, 46, 48, 49, 52, 58] while wanting to control their own excretion [48]. Patients are striving to breathe independently again and change positions on their own. They long for oral food intake [48], water to drink [48, 50, 53, 54] and different flavors [52, 53]. Oral care and frequent moistening are considered important interventions [40, 42, 48, 50, 53, 54]. Patients long for comfortable sleep [48, 51, 53], relaxation [48, 51, 53], and sedation when they are too exhausted [52, 55]. “And then just as I got to sleep, someone would always jerk me awake to do something. I wish they could have done it all at the one time and let me get a bit more rest” [51].

### Striving for an understanding of one’s own health status

As a result of various influencing factors and the necessity of adapting to an altered context, patients develop the need to understand their own health status in relation to their surrounding environment. They frequently highlight the value of continuous communication and the relevance of sharing information [42–46, 49–51, 54, 57, 58]. “When somebody asks you a question and how silly it seems to you to answer their question, because no matter how menial it might seem, it might help save their life. It really, really is important” [46]. In this context, it is essential to convey information gradually to avoid overwhelming patients [42]. However, they also need details to understand everything and be able to participate [43, 46]. “I want to know some of the details, because it helps me to figure out better what’s going on” [46]. Seeing and feeling are also key senses for patients under MV to prevent misinterpretation [42, 44]. “And I can remember he said, that it was very important that I didn’t touch it [the tube]. And I could sense on the way he said it, that it was important” [50].

### Longing for personal integrity

During their treatment process, patients develop and maintain a constant need for personal integrity. This is characterized by a strong desire for normality [5, 52, 53, 55], physical and psychological well-being and rapid recovery [52, 53, 56]. Patients want to be involved in decisions [44, 48], demand privacy [44, 47, 51–53, 55, 57, 58] and strive for independence and control [44, 48, 50, 52, 53, 56]. “I even remember once when I was anesthetized. […] [E]ven in the moment of unconsciousness I wanted to control myself to prevent it, but I remember that it happened. Then I was very upset. […] I wanted to cry all the time because I was so sad. I could not say anything” [48].

### Longing for social interaction

Patients under MV also develop a pronounced need for social interaction. This relates not only to HCPs [57], but also to their families and friends [44, 47, 58]. They emphasize the particular importance of visiting opportunities and often look forward to visiting time for hours [47, 52, 53, 57, 59]. The relatives restore their old, normal world to them. “I had forgotten my whole life, my children, and even my husband. When they came there, I found that I had my own home, my husband, and my kids. It was very good and gave me a lot of hope” [47].

Relatives protect patients in the ICU and stand up for them [47, 53, 55, 58]. Many processes are easier with their relatives. They feel better understood and mentally stronger during difficult moments. “I could not stay in the ICU even for one minute, and it was very hard for me. When visitors came once a day just for half an hour, it could help me to bear such difficult conditions” [47]. Finally, patients long for social interaction between HCPs and their families, which is expressed in the wish that their families, like themselves, are well cared for in the ICU [44, 46].

## Finding ways to deal with the situation

Following their differentiated perceptions and partial identification of needs, patients focus on finding strategies to deal with the current situation. On the one hand, they face an unexpected situation and perceptions that they have to cope with, even though they have no specific need to do so. On the other hand, the multitude of perceptions gives rise to various needs, which patients aim to address either independently or with the help of others. Subsequently, they evaluate the respective strategies and reflect on their effects. Finally, patients derive consequences from their entire experience, which they translate into revised assumptions and future prospects. At this point, the patients integrate new insights into their stable picture of reality and the process starts all over again.

### Having to cope internally alone

Through MV and its treatment in the ICU, patients are confronted with a situation in which they have to react immediately to stimuli without having reflected on a corresponding need beforehand. The underlying perception is diffuse and depends on different external stimuli or one’s own bodily sensations. It can suddenly appear, slowly become stronger, or even disappear completely. Patients are sensitive to sound and light [48, 59] and hear HCPs talking around them [40, 41, 44–46, 55, 59]. They perceive time [45, 47–53, 57], assume vomiting [48], have incoherent thoughts [50] and experience hallucinations and nightmares [41, 44, 45, 48, 50–53, 58, 59]. Their perception leaves patients with different impressions and interpretations [41, 44, 49–51, 58, 59]. One interviewee clearly stated: “I was terrified by the personnel’s talks” [40]. Another patient reported: “It was a state that I knew I was in hospital, but I thought I was captured by some people. I wanted to run away from there” [48].

Following this diffuse perception, patients cope with their own subjective reality to varying degrees and in different ways, or they experience external ways of coping. On the one hand, patients think a lot about their illness [44, 48, 53, 59], their relatives and their previous life [4, 48]. They relate conversations about other people to themselves [40, 44, 55] and compare themselves with others [46]. Patients make hasty decisions out of fear [48] or cry out of despair [48]. On the other hand, they are repeatedly told to calm down and relax [40, 43]. Often, they simply have to endure the situation, the intervention or their condition [43, 46, 47, 51, 53]. “I think the best way is once you’ve had it done once and you know you’ve gotta have it done a second time is just to grin and bear it, really attack it mentally otherwise it’d just bloody drive you crazy” [43].

### Trying to satisfy the needs oneself

To satisfy emerging needs, patients under MV develop strategies by themselves. These are characterized by active behavior and can be applied by patients more or less independently. For example, they watch television to bridge the time [53], pull out their catheters due to pain or discomfort [42, 44, 45, 58], or support HCPs in their work [52, 53, 58]. One patient stated that it is “good to do the suctioning oneself in one's mouth. […] Know myself how far down to go” [53]. However, a patient's active behavior is not always only physically and objectively observable, it can also take place mentally. Patients practice accepting their situation [43, 50, 57–59]. They are trying to face and endure their feelings [40, 47, 50, 54, 55, 58, 59] and become mentally strong [40, 43, 49–51, 54, 58]. Holm [50] cites an interviewee who reflects: “Well of course it [the tube] is unpleasant to have, it's no joyride, you know. But when one day had passed, I thought – well then it can stay there for a couple of days more. And when you have managed that, then you can do it."

### Getting one’s needs fulfilled by others

In addition to their own response to needs, patients under MV also receive responses from third parties, intentionally or not. HCPs or relatives perform different activities that address the need specifically or satisfy it incidentally. In practice, patients are being washed, moisturized in the mouth, assisted in their elimination, or moved from side to side in bed by HCPs [48, 54, 56, 58]. Relatives also play an important role and (un)intentionally support patients in their communication [40, 43, 44, 46, 47, 52, 57, 58]. They monitor and protect patients [47, 55] and provide distraction from their situation [44, 58]. “I wasn’t forced to think about the illness continually when she [relative] was with me" [44].

### Evaluating strategies depending on the situation

After (un)targeted, (un)intended coping and need-related interventions, patients assign meaning to the experienced strategies and evaluate them. In their evaluation, patients are not just looking for a simple ‘yes’ or ‘no’ answer to the question of whether the strategies were successful or not. In fact, they look for an answer on a ‘more or less-continuum’ and thereby consider several outcomes. As a result, the evaluation of a strategy is often complex and various interpretations occur simultaneously. For example, patients reported that they experienced pain and nausea from endotracheal suction [48, 51, 53, 58], but that this also provided considerable relief to their breathing [53, 58]. Visiting as an overall positive intervention relieves loneliness and pain [40, 44, 47, 58, 59], and reassures patients [47, 55, 58] but might also trigger discomfort when distant relatives are present [47].

Furthermore, the factor ‘time’ is of key importance in the evaluation of strategies. Patients frequently reported feeling safe because of constant monitoring [52, 55, 58, 59]. After a certain time, many patients feel irritated and disturbed by cables, which is why they keep removing them [48]. At the beginning of their treatment, patients often feel misunderstood, not heard or ignored [42–44, 46–51, 55, 57]. However, they have also increasingly developed an understanding of their situation and alternative methods for functional communication [44, 52, 53, 55, 57, 58].

In addition to these controversies, patients might have a different understanding and evaluation of their situation than others do [40, 48]. A patient reports on a situation in which he was informed by a nurse. The nurse thought she had informed him sufficiently and said nothing more afterwards. However, he was left with uncertainty and more questions than before: “My nurse told [me] to be calm, [as] your test result was received, we would take out that tube […]. I did not know what this test was [for], maybe its result would take several more days to come” [40]. Another patient reported on her self-assessment and external assessment after receiving several interventions: “Suddenly I saw my mom come up in a very good mood. […] She kept laughing. She said: Do not be upset because you are in a very good condition. But I thought: God! Now I am feeling so bad, I cannot talk at all, I cannot move, like a piece of meat. Why is my mom behaving like this?” [48]

### Drawing consequences from the situation

After a comprehensive process of perceiving, dealing and evaluating, patients face their situation based on experience. They review their own assumptions and harmonize them with the actual experience, thereby either retaining or revising them [44, 48, 51]. Patients develop an increasingly comprehensive picture and understanding of their situation, in which not only each individual but also the HCPs and their environment are integrated. On this basis, patients either build up or lose trust in their environment and the HCPs [40, 41, 43, 44, 46, 47, 51–53, 55, 56, 58, 59]. If they experience poor care and feel uncomfortable in their situation, patients become agitated and angry or depressed and introverted [47, 48]. As a result, some patients develop indifferent attitudes [42, 46, 48–51, 53, 55, 58, 59] and suppress their own feelings and needs [50, 53, 54, 57]. A patient explained why he no longer communicated with HCPs: “It didn’t make any difference. They were going to hear what they wanted to hear” [46]. Another patient describes that he no longer resisted suctioning: “So I ended up I gave up. What did it matter how I felt?” [51]

However, patients under MV also develop confidence and hope if they gain positive experiences. The humor and ease of HCPs help them [53, 56], as do frequent visits from relatives [40, 47, 52, 57, 59]. “The presence of support people could give me hope. I was really encouraged. […] I thought that I was good, so my doctor had let my kids come and visit me. I became hopeful” [47]. Patients are also encouraged when they notice recovery and progress [52, 59], become involved in the therapy [52, 55, 56, 58] and gradually overcome their dependence on others [40, 44, 50, 52, 55, 56, 58]. One patient described his regained mobility as follows: “It was nice that they respected it and seemed to think it was good that I did it because then they understood that they didn’t have to apply suction but that I had managed it myself” [52].

## Discussion

### Summary of evidence

The aim of this review was to provide an in-depth analysis and synthesis of qualitative studies on the experience of conscious patients under MV. Therefore, a meta-synthesis was conducted in accordance with the methodological recommendations of Sandelowski and Barroso [25]. In total, we screened 2,563 references and included 20 studies. The synthesis revealed the central phenomenon of patients ‘being an observer of their own lives’. This phenomenon is characterized by three major concepts. Patients are ‘yearning for a stable picture of reality’. They are ‘developing various situation-specific needs’ and ‘finding ways to deal with the situation’.

These concepts and their subcodes were consistently confirmed in all studies and are based on more than 3,000 coded text passages. At this point, our approach clearly differs from existing meta-syntheses on this topic [20–22]. With respect to 20 articles, we included significantly more studies than did previous syntheses. Although it is recommended that fewer studies should be considered in a meta-synthesis due to a possible, superficial analysis [60], we were able to avoid imprecision and ensure high methodological quality. For this purpose, we conducted a differentiated and multifaceted analysis with three reviewers and numerous discussion rounds. As we intended to develop an in-depth understanding of the patient experience, we also included only studies that provided a rich description of their results. For this reason, we excluded several studies from the analysis that provided only a brief or superficial description of their results [e.g.,^61^–^63^] (see Additional file 4). Nonetheless, it should be noted that far more than the 20 studies we included address the experience of patients under MV. In addition, we included only peer-reviewed articles [64], although Sandelowski and Barroso recommended that gray literature and other sources might also be considered [25]. However, we believe that the exclusion of other papers is justified by the existing broad evidence base.

Through our systematic search and additional hand and citation searches, we can assume that we have identified the relevant publications on our topic. The majority of publications originate from Europe and other Western countries. Although this corresponds to the other available meta-syntheses [20–22], it raises the question of why there is no research on this topic from other countries and continents. It is evident that considerably less research has been carried out and published in nonindustrial nations [65]. In addition, the 10/90 gap of the Global Forum for Health Research highlights the fact that 90% of internationally available health research addresses only 10% of global health problems [66, 67]. This health inequity is also evident in the small number of ICUs and limited options for MV treatment in developing countries [68, 69]. Therefore, it remains unclear to what extent the results of this synthesis can be applied to populations other than those included.

With respect to the experience under MV, the evidence illustrates that patients recall their ICU stay increasingly less and more indifferently over time [70]. This was also confirmed in our meta-synthesis as patients described a fluctuating perception of consciousness [41, 45, 47–51, 57–59]. To minimize recall bias, we only included studies that investigated patients within six months after MV, which is in line with current methodological recommendations [29, 30].

In fact, 14 of the 20 studies collected their data immediately after MV. The patients were interviewed while still in the ICU [41, 47–50, 54], a few days after discharge from the ICU [40, 43] or during their hospital stay in the subsequent general ward [42, 45, 52, 53, 55, 56, 59] (see Table 1). Nevertheless, the question remains as to how comprehensively the overall experience of patients under MV could be empirically illustrated to date. Since sedation regimens are changing and patients are increasingly awake under MV [4, 5], their experience is also potentially changing. However, researchers could also identify this as an opportunity to conduct studies on patients’ lived experience already during MV and complete the data.

While our meta-synthesis addresses only the experience of MV in the ICU, it is also important to consider how patients fare after their ICU stay. Current research indicates that delirium and delusional memories in the ICU lead to impaired cognitive function in patients after discharge. They may only partially recall their ICU stay and their experiences under MV [71–75]. In addition, studies have revealed that patients show increased rates of anxiety, depression or posttraumatic stress disorder and that their quality of life is significantly impaired afterwards [76–80]. Since the experience in the ICU is largely responsible for this, targeted interventions are needed at an early stage to prevent negative outcomes. Our meta-synthesis revealed interactions between HCPs and patients as well as information and communication as key interventions to substantially influence the further course and psyche of patients. In addition, tools such as the ICU diary can be used. This has been proven to fill gaps in memory and promote mental recovery [81–85].

The synthesis also highlighted that patients long for normality [52, 53, 58] but are confronted with major changes [40, 43–45, 47, 48, 51, 52, 55, 56, 58, 59]. During their treatment and over time, they adapt their picture of reality based on their experiences [44, 48, 51]; patients are building a new normal and anticipating their future [40, 41, 43–55, 58, 59]. This result is also evident in other studies, e.g. on end-of-life care for patients [86], and emphasizes the need to orient care toward patients’ new understanding of normality to facilitate individualized care. However, there are still insufficient data to provide a comprehensive and in-depth understanding of the underlying process and the development of a new reality. Therefore, further research into these issues is recommended.

### Limitations

Our meta-synthesis has limitations. As we included only studies in English, German or Spanish, publications in other languages were possibly not included. In addition, the data collection and evaluation methods of the included studies differ. The fact that heterogeneous results are brought together in the meta-synthesis can be viewed critically because unequal data are analyzed equally. However, this approach is explicitly recommended by Sandelowski and Barroso [25, 87]. Finally, we carried out a quality appraisal, which did not give rise to any major concerns. As Sandelowski and Barroso left open the extent to which quality influences the synthesis [25], we did not further consider our quality appraisal. Nevertheless, we restricted the inclusion criteria around recall bias, rich description and peer review to identify and include only studies relevant to our research question.

## Conclusion

This meta-synthesis provides insights into the perception of intensive care patients under MV who are continuously observing their own lives. This process is complex and involves a highly differentiated perception of their environment, the categorization and interpretation of all sensory inputs, as well as the development of situation-specific needs and ways of dealing with them. This cyclical process is ongoing and includes interaction with HCPs and the care they provide.

Due to the potentially negative long-term effects of an ICU stay for patients and the unique responsibility of HCPs in interactions and as advocates for patients, it is of great importance that they are constantly sensitive to the perceptiveness and differentiated experience of patients under MV. After all, positive outcomes such as faster weaning can be achieved in particular by knowing their patients and tailoring the therapy to their experience.

## Supplementary Information

Additional file1 (PDF 301 KB)

Additional file2 (PDF 395 KB)

Additional file3 (PDF 214 KB)

Additional file4 (PDF 133 KB)

## Data Availability

The datasets supporting the conclusions of this article are included within the article and its additional files. Further data used and/or analyzed during the current study are available from the corresponding author.

## List of abbreviations

CASP: Critical appraisal skills programme
CINAHL: Cumulative index to nursing and allied health sciences
ENTREQ: Enhancing transparency in reporting the synthesis of qualitative research
HCP: Healthcare professional
ICU: Intensive care unit
MV: Mechanical ventilation
PICo: Population, phenomenon of interest, context
PRISMA: Preferred reporting items for systematic reviews and meta-analyses
