# Supplementary material for: Being an observer of one’s own life—a meta-synthesis on the experience of mechanically ventilated patients in intensive care units
Source: Crit Care. 2025 Mar 8;29:105. doi: 10.1186/s13054-025-05326-6 (PMC11889880; doi:10.1186/s13054-025-05326-6)
Supplement: Supplementary file 1 — Additional file1 (PDF 301 KB) [file 13054_2025_5326_MOESM1_ESM.pdf]

Additional file 1: Search strategies of the meta-synthesis

1 - Systematic searches within databases

Meta-synthesis on the experience of ventilated inpatients during weaning

**Medline (via PubMed)**<br>Date of search: 23.01.2024			
**Content**	**Number**	**Keyword(s)**	**Results**
**Ventilator weaning/ artificial respiration**	#1	artificial respiration[MeSH Terms]	89,965
	#2	mechanical* ventilat*[Title/Abstract]	79,790
	#3	invasive* ventilat*[Title/Abstract]	19,339
	#4	artificial* ventilat*[Title/Abstract]	47,493
	#5	invasive* respir*[Title/Abstract]	21,144
	#6	ventilator weaning[MeSH Terms]	4,520
	#7	ventilator weaning[Title/Abstract]	762
	#8	ventilator liberat*[Title/Abstract]	88
	#9	airway extubation[MeSH Terms]	2,488
	#10	extubat*[Title/Abstract]	17,494
	#11	decannulat*[Title/Abstract]	3,230
	**#12**	**#1-11/OR**<br>(((((((((artificial respiration[MeSH Terms]) OR (mechanical* ventilat*[Title/Abstract])) OR (invasive* ventilat*[Title/Abstract])) OR (artificial* ventilat*[Title/Abstract])) OR (invasive* respir*[Title/Abstract])) OR (ventilator weaning[MeSH Terms])) OR (ventilator weaning[Title/Abstract])) OR (ventilator liberat*[Title/Abstract])) OR (airway extubation[MeSH Terms])) OR (extubat*[Title/Abstract])) OR (decannulat*[Title/Abstract])	**172,956**
**Experience**	#13	perception[MeSH Terms]	484,432
	#14	percept*[Title/Abstract]	404,499
	#15	awareness[MeSH Terms]	22,153
	#16	aware*[Title/Abstract]	315,298
	#17	experienc*[Title/Abstract]	1,464,746
	#18	feel*[Title/Abstract]	125,624
	#19	emotions[MeSH Terms]	419,996
	#20	emotion*[Title/Abstract]	273,811
	**#21**	**#13-20/OR**<br>(((((((perception[MeSH Terms]) OR (percept*[Title/Abstract])) OR (awareness[MeSH Terms])) OR (aware*[Title/Abstract])) OR (experienc*[Title/Abstract])) OR (feel*[Title/Abstract])) OR (emotions[MeSH Terms])) OR (emotion*[Title/Abstract])	**2,854,718**
**Qualitative research**	#22	case study[Title/Abstract]	103,397
	#23	constant comparison analysis[Title/Abstract]	123
	#24	content analysis[Title/Abstract]	45,754
	#25	conversation analysis[Title/Abstract]	967
	#26	descriptive study[Title/Abstract]	36,857
	#27	discourse analysis[Title/Abstract]	2,675
	#28	Ethnography[Title/Abstract]	4,818
	#29	Exploratory[Title/Abstract]	108,684
	#30	field observation[Title/Abstract]	822
	#31	field study[Title/Abstract]	10,159
	#32	focus group[Title/Abstract]	36,661
	#33	grounded theory[Title/Abstract]	15,660
	#34	Hermeneutic[Title/Abstract]	3,464
	#35	interview study[Title/Abstract]	5,803

	#36	interview*[Title/Abstract]	470,753
	#37	narrative analysis[Title/Abstract]	1,921
	#38	naturalistic inquiry[Title/Abstract]	225
	#39	participant observation[Title/Abstract]	4,245
	#40	Phenomenology[Title/Abstract]	12,094
	#41	qualitative research[MeSH Terms]	85,422
	#42	qualitativ*[Title/Abstract]	396,594
	#43	Semiotics[Title/Abstract]	623
	#44	semiotic analysis[Title/Abstract]	47
	#45	thematic analysis[Title/Abstract]	43,159
	**#46**	**#22-#45/OR**<br>((((((((((((((((((((((Case study[Title/Abstract]) OR (Constant comparison analysis[Title/Abstract])) OR (Content analysis[Title/Abstract])) OR (Conversation analysis[Title/Abstract])) OR (Descriptive study[Title/Abstract])) OR (discourse analysis[Title/Abstract])) OR (Ethnography[Title/Abstract])) OR (Exploratory[Title/Abstract])) OR (Field observation[Title/Abstract])) OR (Field study[Title/Abstract])) OR (Focus group[Title/Abstract])) OR (Grounded theory[Title/Abstract])) OR (Hermeneutic[Title/Abstract])) OR (interview study[Title/Abstract])) OR (narrative analysis[Title/Abstract])) OR (Naturalistic inquiry[Title/Abstract])) OR (Participant observation[Title/Abstract])) OR (Phenomenology[Title/Abstract])) OR (qualitative research[MeSH Terms])) OR (qualitativ*[Title/Abstract])) OR (Semiotics[Title/Abstract])) OR (semiotic analysis[Title/Abstract])) OR (Thematic analysis[Title/Abstract])) OR (interview*[Title/Abstract])	**1,006,478**
**All combined**	**#47**	**#12 AND #21 AND #46**<br>(((((((((((artificial respiration[MeSH Terms]) OR (mechanical* ventilat*[Title/Abstract])) OR (invasive* ventilat*[Title/Abstract])) OR (artificial* ventilat*[Title/Abstract])) OR (invasive* respir*[Title/Abstract])) OR (ventilator weaning[MeSH Terms])) OR (ventilator weaning[Title/Abstract])) OR (ventilator liberat*[Title/Abstract])) OR (airway extubation[MeSH Terms])) OR (extubat*[Title/Abstract])) OR (decannulat*[Title/Abstract])) AND ((((((((perception[MeSH Terms]) OR (percept*[Title/Abstract])) OR (awareness[MeSH Terms])) OR (aware*[Title/Abstract])) OR (experienc*[Title/Abstract])) OR (feel*[Title/Abstract])) OR (emotions[MeSH Terms])) OR (emotion*[Title/Abstract]))) AND (((((((((((((((((((((((Case study[Title/Abstract]) OR (Constant comparison analysis[Title/Abstract])) OR (Content analysis[Title/Abstract])) OR (Conversation analysis[Title/Abstract])) OR (Descriptive study[Title/Abstract])) OR (discourse analysis[Title/Abstract])) OR (Ethnography[Title/Abstract])) OR (Exploratory[Title/Abstract])) OR (Field observation[Title/Abstract])) OR (Field study[Title/Abstract])) OR (Focus group[Title/Abstract])) OR (Grounded theory[Title/Abstract])) OR (Hermeneutic[Title/Abstract])) OR (interview study[Title/Abstract])) OR (narrative analysis[Title/Abstract])) OR (Naturalistic inquiry[Title/Abstract])) OR (Participant observation[Title/Abstract])) OR (Phenomenology[Title/Abstract])) OR (qualitative research[MeSH Terms])) OR (qualitativ*[Title/Abstract])) OR (Semiotics[Title/Abstract])) OR (semiotic analysis[Title/Abstract])) OR (Thematic analysis[Title/Abstract])) OR (interview*[Title/Abstract]))	**1,014**

Cochrane Library<br>Date of search: 23.01.2024			
**Content**	**Number**	**Keyword(s)**	**Results**
**Ventilator weaning/ artificial respiration**	#1	artificial respiration[MeSH descriptor]	8,357
	#2	mechanical* ventilat*[Title/Abstract/Keyword]	16,364
	#3	invasive* ventilat*[Title/Abstract/Keyword]	5,880
	#4	artificial* ventilat*[Title/Abstract/Keyword]	11,358
	#5	invasive* respir*[Title/Abstract/Keyword]	5,464
	#6	ventilator weaning[MeSH descriptor]	629
	#7	ventilator weaning[Title/Abstract/Keyword]	1,446
	#8	ventilator liberat*[Title/Abstract/Keyword]	103
	#9	airway extubation[MeSH descriptor]	350
	#10	extubat*[Title/Abstract/Keyword]	10,062
	#11	decannulat*[Title/Abstract/Keyword]	161
	**#12**	**#1-11/OR**<br>artificial respiration[MeSH descriptor] OR mechanical* ventilat*[Title/Abstract/Keyword] OR invasive* ventilat*[Title/Abstract/Keyword] OR artificial* ventilat*[Title/Abstract/Keyword] OR invasive* respir*[Title/Abstract/Keyword] OR ventilator weaning[MeSH descriptor] OR ventilator weaning[Title/Abstract/Keyword] OR ventilator liberat*[Title/Abstract/Keyword] OR airway extubation[MeSH descriptor] OR extubat*[Title/Abstract/Keyword] OR decannulat*[Title/Abstract/Keyword]	**35,405**
**Experience**	#13	perception[MeSH descriptor]	23,010
	#14	percept*[Title/Abstract/Keyword]	41,860
	#15	awareness[MeSH descriptor]	1,481
	#16	aware*[Title/Abstract/Keyword]	19,931
	#17	experienc*[Title/Abstract/Keyword]	143,869
	#18	feel*[Title/Abstract/Keyword]	19,112
	#19	emotions[MeSH descriptor]	40,227
	#20	emotion*[Title/Abstract/Keyword]	37,077
	**#21**	**#13-20/OR**<br>perception[MeSH descriptor] OR percept*[Title/Abstract/Keyword] OR awareness[MeSH descriptor] OR aware*[Title/Abstract/Keyword] OR experienc*[Title/Abstract/Keyword] OR feel*[Title/Abstract/Keyword] OR emotions[MeSH descriptor] OR emotion*[Title/Abstract/Keyword]	**263,782**
**Qualitative research**	#22	case study[Title/Abstract/Keyword]	65,713
	#23	constant comparison analysis[Title/Abstract/Keyword]	749
	#24	content analysis[Title/Abstract/Keyword]	13,909
	#25	conversation analysis[Title/Abstract/Keyword]	833
	#26	descriptive study[Title/Abstract/Keyword]	26,910
	#27	discourse analysis[Title/Abstract/Keyword]	153
	#28	ethnography[Title/Abstract/Keyword]	65
	#29	exploratory[Title/Abstract/Keyword]	26,402
	#30	field observation[Title/Abstract/Keyword]	873
	#31	field study[Title/Abstract/Keyword]	21,446
	#32	focus group[Title/Abstract/Keyword]	17,827
	#33	grounded theory[Title/Abstract/Keyword]	753
	#34	hermeneutic*[Title/Abstract/Keyword]	38
	#35	interview study[Title/Abstract/Keyword]	24,839
	#36	interview*[Title/Abstract/Keyword]	50,083
	#37	narrative analysis[Title/Abstract/Keyword]	1,231
	#38	naturalistic inquiry[Title/Abstract/Keyword]	33
	#39	participant observation[Title/Abstract/Keyword]	2,061
	#40	Phenomenology[Title/Abstract/Keyword]	209
	#41	qualitative research[MeSH descriptor]	2,095
	#42	qualitativ*[Title/Abstract/Keyword]	23,874
	#43	Semiotics[Title/Abstract/Keyword]	4
	#44	semiotic analysis[Title/Abstract/Keyword]	2
	#45	thematic analysis[Title/Abstract/Keyword]	2,927

	**#46**	**#22-#45/OR**<br>case study[Title/Abstract/Keyword] OR constant comparison analysis[Title/Abstract/Keyword] OR content analysis[Title/Abstract/Keyword] OR conversation analysis[Title/Abstract/Keyword] OR descriptive study[Title/Abstract/Keyword] OR discourse analysis[Title/Abstract/Keyword] OR ethnography[Title/Abstract/Keyword] OR exploratory[Title/Abstract/Keyword] OR field observation[Title/Abstract/Keyword] OR field study[Title/Abstract/Keyword] OR focus group[Title/Abstract/Keyword] OR grounded theory[Title/Abstract/Keyword] OR hermeneutic*[Title/Abstract/Keyword] OR interview study[Title/Abstract/Keyword] OR interview*[Title/Abstract/Keyword] OR narrative analysis[Title/Abstract/Keyword] OR naturalistic inquiry[Title/Abstract/Keyword] OR participant observation[Title/Abstract/Keyword] OR Phenomenology[Title/Abstract/Keyword] OR qualitative research[MeSH descriptor] OR qualitativ*[Title/Abstract/Keyword] OR Semiotics[Title/Abstract/Keyword] OR semiotic analysis[Title/Abstract/Keyword] OR thematic analysis[Title/Abstract/Keyword]	**212,156**
**All combined**	**#47**	**#12 AND #21 AND #46**<br>(artificial respiration[MeSH descriptor] OR mechanical* ventilat*[Title/Abstract/Keyword] OR invasive* ventilat*[Title/Abstract/Keyword] OR artificial* ventilat*[Title/Abstract/Keyword] OR invasive* respir*[Title/Abstract/Keyword] OR ventilator weaning[MeSH descriptor] OR ventilator weaning[Title/Abstract/Keyword] OR ventilator liberat*[Title/Abstract/Keyword] OR airway extubation[MeSH descriptor] OR extubat*[Title/Abstract/Keyword] OR decannulat*[Title/Abstract/Keyword]) AND (perception[MeSH descriptor] OR percept*[Title/Abstract/Keyword] OR awareness[MeSH descriptor] OR aware*[Title/Abstract/Keyword] OR experienc*[Title/Abstract/Keyword] OR feel*[Title/Abstract/Keyword] OR emotions[MeSH descriptor] OR emotion*[Title/Abstract/Keyword]) AND (case study[Title/Abstract/Keyword] OR constant comparison analysis[Title/Abstract/Keyword] OR content analysis[Title/Abstract/Keyword] OR conversation analysis[Title/Abstract/Keyword] OR descriptive study[Title/Abstract/Keyword] OR discourse analysis[Title/Abstract/Keyword] OR ethnography[Title/Abstract/Keyword] OR exploratory[Title/Abstract/Keyword] OR field observation[Title/Abstract/Keyword] OR field study[Title/Abstract/Keyword] OR focus group[Title/Abstract/Keyword] OR grounded theory[Title/Abstract/Keyword] OR hermeneutic*[Title/Abstract/Keyword] OR interview study[Title/Abstract/Keyword] OR interview*[Title/Abstract/Keyword] OR narrative analysis[Title/Abstract/Keyword] OR naturalistic inquiry[Title/Abstract/Keyword] OR participant observation[Title/Abstract/Keyword] OR Phenomenology[Title/Abstract/Keyword] OR qualitative research[MeSH descriptor] OR qualitativ*[Title/Abstract/Keyword] OR Semiotics[Title/Abstract/Keyword] OR semiotic analysis[Title/Abstract/Keyword] OR thematic analysis[Title/Abstract/Keyword])	**686**

**CINAHL**<br>Date of search: 23.01.2024			
**Content**	**Number**	**Keyword(s)**	**Results**
**Ventilator weaning/ artificial respiration**	#1	(MH "Respiration, Artificial")	25,916
	#2	mechanical* ventilat*	26,960
	#3	invasive* ventilat*	4,366
	#4	artificial* ventilat*	614
	#5	invasive* respir*	922
	#6	(MH "Ventilator Weaning")	2,940
	#7	ventilator weaning	3,140
	#8	ventilator liberat*	105
	#9	(MH "Extubation")	1,416
	#10	extubat*	6,008
	#11	decannulat*	806
	**#12**	**#1-11/OR**<br>(MH "Respiration, Artificial") OR mechanical* ventilat* OR invasive* ventilat* OR artificial* ventilat* OR invasive* respir* OR (MH "Ventilator Weaning") OR ventilator weaning OR ventilator liberat* OR (MH "Extubation") OR extubat* OR decannulat*	**47,660**
**Experience**	#13	(MH "Perception")	31,164
	#14	percept*	205,280
	#15	(MH "Cognition")	69,831
	#16	aware*	131,846
	#17	experienc*	600,173
	#18	feel*	73,204
	#19	(MH "Emotions")	46,819
	#20	emotion*	143,673
	**#21**	**#13-20/OR**<br>(MH "Perception") OR percept* OR (MH "Cognition") OR aware* OR experienc* OR feel* OR (MH "Emotions") OR emotion*	**1,014,773**
**Qualitative research**	#22	case study	223,572
	#23	constant comparison analysis	655
	#24	content analysis	59,487
	#25	conversation analysis	1,230
	#26	descriptive study	64,750
	#27	discourse analysis	6,858
	#28	ethnography	3,814
	#29	exploratory	78,327
	#30	field observation	1,802
	#31	field study	14,032
	#32	focus group	66,369
	#33	grounded theory	22,523
	#34	hermeneutic*	5,119
	#35	interview study	22,640
	#36	interview*	390,485
	#37	narrative analysis	4,866
	#38	naturalistic inquiry	11,945
	#39	participant observation	10,042
	#40	Phenomenology	8,415
	#41	(MH "Qualitative Studies")	150,534
	#42	qualitativ*	235,464
	#43	Semiotics	317
	#44	semiotic analysis	60
	#45	thematic analysis	102,209
	**#46**	**#22-#45/OR**<br>case study OR constant comparison analysis OR content analysis OR conversation analysis OR descriptive study OR discourse analysis OR ethnography OR exploratory OR field observation OR field study OR focus group OR grounded theory OR hermeneutic* OR interview study OR interview* OR narrative analysis OR naturalistic inquiry OR participant observation OR Phenomenology OR (MH "Qualitative	**832,909**

		Studies") OR qualitativ* OR Semiotics OR semiotic analysis OR thematic analysis	
**All combined**	**#47**	**#12 AND #21 AND #46**<br>((MH "Respiration, Artificial") OR mechanical* ventilat* OR invasive* ventilat* OR artificial* ventilat* OR invasive* respir* OR (MH "Ventilator Weaning") OR ventilator weaning OR ventilator liberat* OR (MH "Extubation") OR extubat* OR decannulat*) AND ((MH "Perception") OR percept* OR (MH "Cognition") OR aware* OR experienc* OR feel* OR (MH "Emotions") OR emotion*) AND (case study OR constant comparison analysis OR content analysis OR conversation analysis OR descriptive study OR discourse analysis OR ethnography OR exploratory OR field observation OR field study OR focus group OR grounded theory OR hermeneutic* OR interview study OR interview* OR narrative analysis OR naturalistic inquiry OR participant observation OR Phenomenology OR (MH "Qualitative Studies") OR qualitativ* OR Semiotics OR semiotic analysis OR thematic analysis)	**848**

2 - Additional hand search

Source	Search term	Filter	Date of search	Relevant results
LIVIVO	Experience mechanical ventilation	None	26.05.2024	1
	Experiencing mechanical ventilation	None	26.05.2024	
	Experience artificial respiration	None	26.05.2024	
	Experiencing artificial respiration	None	26.05.2024	
	Experience ventilator weaning	None	26.05.2024	
	Experiencing ventilator weaning	None	26.05.2024	
	Experience respirator support	None	26.05.2024	
	Experiencing respirator support	None	26.05.2024	
	Perception mechanical ventilation	None	26.05.2024	
	Perceive mechanical ventilation	None	26.05.2024	
	Perception artificial respiration	None	27.05.2024	
	Perceive artificial respiration	None	27.05.2024	
	Perception ventilator weaning	None	27.05.2024	
	Perceive ventilator weaning	None	27.05.2024	
	Perception respirator support	None	27.05.2024	
	Perceive respirator support	None	27.05.2024	
Google Scholar	Experience mechanical ventilation	None	27.05.2024	4
	Experiencing mechanical ventilation	None	27.05.2024	
	Experience artificial respiration	None	27.05.2024	
	Experiencing artificial respiration	None	27.05.2024	
	Experience ventilator weaning	None	27.05.2024	
	Experiencing ventilator weaning	None	27.05.2024	
	Experience respirator support	None	28.05.2024	
	Experiencing respirator support	None	28.05.2024	
	Perception mechanical ventilation	None	28.05.2024	
	Perceive mechanical ventilation	None	28.05.2024	
	Perception artificial respiration	None	28.05.2024	
	Perceive artificial respiration	None	28.05.2024	
	Perception ventilator weaning	None	28.05.2024	
	Perceive ventilator weaning	None	28.05.2024	
	Perception respirator support	None	28.05.2024	
	Perceive respirator support	None	28.05.2024	

3 - Citation searching

Relevant studies: **10**

Seed references for citation searching:

- Baumgarten, M., & Poulsen, I. (2015). Patients' experiences of being mechanically ventilated in an ICU: a qualitative metasynthesis. *Scandinavian journal of caring sciences, 29*(2), 205–214. https://doi.org/10.1111/scs.12177
- Carroll S. M. (2004). Nonvocal ventilated patients perceptions of being understood. *Western journal of nursing research, 26*(1), 85–112. https://doi.org/10.1177/0193945903259462
- Carruthers, H., Gomersall, T., & Astin, F. (2018). The work undertaken by mechanically ventilated patients in Intensive Care: A qualitative meta-ethnography of survivors' experiences. *International journal of nursing studies, 86*, 60–73. https://doi.org/10.1016/j.ijnurstu.2018.05.013
- Cook, D. J., Meade, M. O., & Perry, A. G. (2001). Qualitative studies on the patient's experience of weaning from mechanical ventilation. *Chest, 120*(6 Suppl), 469S–73S. https://doi.org/10.1378/chest.120.6_suppl.469s
- Danielis, M., Povoli, A., Mattiussi, E., & Palese, A. (2020). Understanding patients' experiences of being mechanically ventilated in the Intensive Care Unit: Findings from a meta-synthesis and meta-summary. *Journal of clinical nursing, 29*(13-14), 2107–2124. https://doi.org/10.1111/jocn.15259
- Egerod, I., Bergbom, I., Lindahl, B., Henricson, M., Granberg-Axell, A., & Storli, S. L. (2015). The patient experience of intensive care: a meta-synthesis of Nordic studies. *International journal of nursing studies, 52*(8), 1354–1361. https://doi.org/10.1016/j.ijnurstu.2015.04.017
- Rose, L., Dainty, K. N., Jordan, J., & Blackwood, B. (2014). Weaning from mechanical ventilation: a scoping review of qualitative studies. *American journal of critical care : an official publication, American Association of Critical-Care Nurses, 23*(5), e54–e70. https://doi.org/10.4037/ajcc2014539
- Tolotti, A., Cadorin, L., Bonetti, L., Valcarenghi, D., & Pagnucci, N. (2023). Communication experiences of tracheostomy patients with nurses in the ICU: A scoping review. *Journal of clinical nursing, 32*(11-12), 2361–2370. https://doi.org/10.1111/jocn.16296
- Tsay, S. F., Mu, P. F., Lin, S., Wang, K. W., & Chen, Y. C. (2013). The experiences of adult ventilator-dependent patients: a meta-synthesis review. *Nursing & health sciences, 15*(4), 525–533. https://doi.org/10.1111/nhs.12049
