# Supplementary material for: Being an observer of one’s own life—a meta-synthesis on the experience of mechanically ventilated patients in intensive care units
Source: Crit Care. 2025 Mar 8;29:105. doi: 10.1186/s13054-025-05326-6 (PMC11889880; doi:10.1186/s13054-025-05326-6)
Supplement: Supplementary file 2 — Additional file2 (PDF 395 KB) [file 13054_2025_5326_MOESM2_ESM.pdf]

Additional file 2: CASP Appraisal of included studies

Meta-synthesis on the experience of patients undergoing mechanical ventilation

Checklist from: Critical Appraisal Skills Programme. (2018). CASP Qualitative Checklist. Available at https://casp-uk.net/casp-tools-checklists/. Accessed at 04/29/2024.

**Aslani et al., 2017**				
Question	**Yes**	**Can't tell**	**No**	**Comments**
Section A: Are the results valid?				
**01. Was there a clear statement of the aims of the research?**	X			
**02. Is a qualitative methodology appropriate?**	X			
**03. Was the research design appropriate to address the aims of the research?**	X			
**04. Was the recruitment strategy appropriate to the aims of the research?**	X			
**05. Was the data collected in a way that addressed the research issue?**	X			
**06. Has the relationship between researcher and participants been adequately considered?**			X	Only information on first contacts, but no details on the relationship
Section B: What are the results?				
**07. Have ethical issues been taken into consideration?**	X			
**08. Was the data analysis sufficiently rigorous?**	X			
**09. Is there a clear statement of findings?**	X			
Section C: Will the results help locally?				
**10. How valuable is the research?**	The study highlights the psychological impact of mechanical ventilation and the sensibility for patients' needs. It discusses its results with other studies and recommends certain aspects to clinicians.			

Ballard et al., 2006				
Question	**Yes**	**Can't tell**	**No**	**Comments**
Section A: Are the results valid?				
**01. Was there a clear statement of the aims of the research?**	X			
**02. Is a qualitative methodology appropriate?**	X			
**03. Was the research design appropriate to address the aims of the research?**	X			
**04. Was the recruitment strategy appropriate to the aims of the research?**	X			
**05. Was the data collected in a way that addressed the research issue?**	X			
**06. Has the relationship between researcher and participants been adequately considered?**			X	No information provided.
Section B: What are the results?				
**07. Have ethical issues been taken into consideration?**	X			
**08. Was the data analysis sufficiently rigorous?**	X			
**09. Is there a clear statement of findings?**	X			
Section C: Will the results help locally?				
**10. How valuable is the research?**	Research explores a new field, that has not been investigated yet. Results are discussed with other studies, recommendations are made for clinicians.			

Dale et al., 2020				
Question	**Yes**	**Can't tell**	**No**	**Comments**
Section A: Are the results valid?				
**01. Was there a clear statement of the aims of the research?**	X			
**02. Is a qualitative methodology appropriate?**	X			
**03. Was the research design appropriate to address the aims of the research?**	X			
**04. Was the recruitment strategy appropriate to the aims of the research?**	X			
**05. Was the data collected in a way that addressed the research issue?**	X			
**06. Has the relationship between researcher and participants been adequately considered?**			X	No information provided.
Section B: What are the results?				
**07. Have ethical issues been taken into consideration?**	X			
**08. Was the data analysis sufficiently rigorous?**	X			
**09. Is there a clear statement of findings?**	X			
Section C: Will the results help locally?				
**10. How valuable is the research?**	Creative research combining interview methods and object elicitation (e.g. tube) to provide in-depth insights. Results are discussed with other studies, limitations are reflected and conclusions are drawn.			

Donnelly et al., 2006				
Question	**Yes**	**Can’t tell**	**No**	**Comments**
Section A: Are the results valid?				
**01. Was there a clear statement of the aims of the research?**	X			
**02. Is a qualitative methodology appropriate?**	X			
**03. Was the research design appropriate to address the aims of the research?**	X			
**04. Was the recruitment strategy appropriate to the aims of the research?**		X		Eligibility criteria are depicted, but no further information on the recruitment strategy
**05. Was the data collected in a way that addressed the research issue?**	X			
**06. Has the relationship between researcher and participants been adequately considered?**		X		A description is presented in parts by reflecting on methods of distanciation.
Section B: What are the results?				
**07. Have ethical issues been taken into consideration?**	X			
**08. Was the data analysis sufficiently rigorous?**	X			
**09. Is there a clear statement of findings?**	X			
Section C: Will the results help locally?				
**10. How valuable is the research?**	Methodological reflection, but no discussion of results with other studies; limitations also reflect methods only.			

Engström et al., 2013				
Question	**Yes**	**Can't tell**	**No**	**Comments**
Section A: Are the results valid?				
**01. Was there a clear statement of the aims of the research?**	**X**			
**02. Is a qualitative methodology appropriate?**	**X**			
**03. Was the research design appropriate to address the aims of the research?**	**X**			
**04. Was the recruitment strategy appropriate to the aims of the research?**	**X**			
**05. Was the data collected in a way that addressed the research issue?**	**X**			
**06. Has the relationship between researcher and participants been adequately considered?**			**X**	
Section B: What are the results?				
**07. Have ethical issues been taken into consideration?**	**X**			
**08. Was the data analysis sufficiently rigorous?**	**X**			
**09. Is there a clear statement of findings?**	**X**			
Section C: Will the results help locally?				
**10. How valuable is the research?**	Rich discussion comparing own results with other studies, own limitations are reflected and conclusions are drawn.			

Gilder et al., 2021				
Question	**Yes**	**Can't tell**	**No**	**Comments**
Section A: Are the results valid?				
**01. Was there a clear statement of the aims of the research?**	**X**			
**02. Is a qualitative methodology appropriate?**	**X**			
**03. Was the research design appropriate to address the aims of the research?**	**X**			
**04. Was the recruitment strategy appropriate to the aims of the research?**	**X**			
**05. Was the data collected in a way that addressed the research issue?**	**X**			
**06. Has the relationship between researcher and participants been adequately considered?**	**X**			
Section B: What are the results?				
**07. Have ethical issues been taken into consideration?**	**X**			
**08. Was the data analysis sufficiently rigorous?**	**X**			
**09. Is there a clear statement of findings?**	**X**			
Section C: Will the results help locally?				
**10. How valuable is the research?**	Broad discussion of own results with other studies, limitations are highlighted and conclusions are drawn.			

**Guttormson et al., 2015**				
Question	**Yes**	**Can't tell**	**No**	**Comments**
Section A: Are the results valid?				
**01. Was there a clear statement of the aims of the research?**	**X**			
**02. Is a qualitative methodology appropriate?**	**X**			
**03. Was the research design appropriate to address the aims of the research?**		**X**		Study is based on a secondary data analysis
**04. Was the recruitment strategy appropriate to the aims of the research?**		**X**		Setting and sample is described, but recruitment strategy lacks clarity.
**05. Was the data collected in a way that addressed the research issue?**			**X**	Next to a questionnaire, authors used three open-ended questions, that are in parts suggestive; the three interview questions do not sufficiently cover the broad research question.
**06. Has the relationship between researcher and participants been adequately considered?**			**X**	No information provided.
Section B: What are the results?				
**07. Have ethical issues been taken into consideration?**	**X**			
**08. Was the data analysis sufficiently rigorous?**	**X**			
**09. Is there a clear statement of findings?**	**X**			
Section C: Will the results help locally?				
**10. How valuable is the research?**	Results are discussed with other studies, short information on limitations is provided, conclusions are short, but in line with study results.			

Hajiabadi et al., 2017				
Question	Yes	Can't tell	No	Comments
Section A: Are the results valid?				
**01. Was there a clear statement of the aims of the research?**	X			
**02. Is a qualitative methodology appropriate?**	X			
**03. Was the research design appropriate to address the aims of the research?**	X			
**04. Was the recruitment strategy appropriate to the aims of the research?**	X			
**05. Was the data collected in a way that addressed the research issue?**	X			
**06. Has the relationship between researcher and participants been adequately considered?**	X			
Section B: What are the results?				
**07. Have ethical issues been taken into consideration?**	X			
**08. Was the data analysis sufficiently rigorous?**	X			
**09. Is there a clear statement of findings?**	X			
Section C: Will the results help locally?				
**10. How valuable is the research?**	Broad discussion of results with other studies, limitations are not sufficiently presented and lacking other potential biases; implications for practice are formulated.			

Hajiabadi et al., 2018				
Question	Yes	Can't tell	No	Comments
Section A: Are the results valid?				
**01. Was there a clear statement of the aims of the research?**	X			
**02. Is a qualitative methodology appropriate?**	X			
**03. Was the research design appropriate to address the aims of the research?**	X			
**04. Was the recruitment strategy appropriate to the aims of the research?**	X			
**05. Was the data collected in a way that addressed the research issue?**	X			
**06. Has the relationship between researcher and participants been adequately considered?**	X			
Section B: What are the results?				
**07. Have ethical issues been taken into consideration?**	X			
**08. Was the data analysis sufficiently rigorous?**	X			
**09. Is there a clear statement of findings?**	X			
Section C: Will the results help locally?				
**10. How valuable is the research?**	Broad discussion of results with other studies, sufficient reflection of limitations, conclusions are drawn.			

Holm et al., 2015				
Question	**Yes**	**Can't tell**	**No**	**Comments**
Section A: Are the results valid?				
**01. Was there a clear statement of the aims of the research?**	X			
**02. Is a qualitative methodology appropriate?**	X			
**03. Was the research design appropriate to address the aims of the research?**	X			
**04. Was the recruitment strategy appropriate to the aims of the research?**	X			
**05. Was the data collected in a way that addressed the research issue?**	X			
**06. Has the relationship between researcher and participants been adequately considered?**		X		Implicit information by giving insights how the research was conducted and the interviews were led, but no specific information.
Section B: What are the results?				
**07. Have ethical issues been taken into consideration?**	X			
**08. Was the data analysis sufficiently rigorous?**	X			
**09. Is there a clear statement of findings?**	X			
Section C: Will the results help locally?				
**10. How valuable is the research?**	Broad discussion of findings with other studies, limitations are subsumed under methodological considerations, conclusions and implications are formulated.			

**Holm et al., 2017**				
Question	**Yes**	**Can't tell**	**No**	**Comments**
Section A: Are the results valid?				
**01. Was there a clear statement of the aims of the research?**	**X**			
**02. Is a qualitative methodology appropriate?**	**X**			
**03. Was the research design appropriate to address the aims of the research?**	**X**			
**04. Was the recruitment strategy appropriate to the aims of the research?**	**X**			
**05. Was the data collected in a way that addressed the research issue?**	**X**			
**06. Has the relationship between researcher and participants been adequately considered?**		**X**		Implicit information by the description of the research process; clear information is lacking.
Section B: What are the results?				
**07. Have ethical issues been taken into consideration?**	**X**			
**08. Was the data analysis sufficiently rigorous?**	**X**			
**09. Is there a clear statement of findings?**	**X**			
Section C: Will the results help locally?				
**10. How valuable is the research?**	Broad discussion of findings with other studies, limitations are reflected as well as the rigour of the study, implications and recommendations for practice are presented and conclusions are drawn.			

Johnson et al., 2006				
Question	**Yes**	**Can't tell**	**No**	**Comments**
Section A: Are the results valid?				
**01. Was there a clear statement of the aims of the research?**	X			
**02. Is a qualitative methodology appropriate?**	X			
**03. Was the research design appropriate to address the aims of the research?**	X			
**04. Was the recruitment strategy appropriate to the aims of the research?**	X			
**05. Was the data collected in a way that addressed the research issue?**	X			
**06. Has the relationship between researcher and participants been adequately considered?**			X	No information provided.
Section B: What are the results?				
**07. Have ethical issues been taken into consideration?**	X			
**08. Was the data analysis sufficiently rigorous?**	X			
**09. Is there a clear statement of findings?**	X			
Section C: Will the results help locally?				
**10. How valuable is the research?**	The focus of the discussion is not comprehensible in some places, e.g. the pathogenesis of delirium or confusion is discussed. No limitations are given, but conclusions can be found.			

Karlsson et al., 2012 A				
Question	**Yes**	**Can't tell**	**No**	**Comments**
Section A: Are the results valid?				
**01. Was there a clear statement of the aims of the research?**	**X**			
**02. Is a qualitative methodology appropriate?**	**X**			
**03. Was the research design appropriate to address the aims of the research?**	**X**			
**04. Was the recruitment strategy appropriate to the aims of the research?**		**X**		Recruitment strategy lacks information on the recruiters and their relationship to patients.
**05. Was the data collected in a way that addressed the research issue?**	**X**			
**06. Has the relationship between researcher and participants been adequately considered?**			**X**	No information provided.
Section B: What are the results?				
**07. Have ethical issues been taken into consideration?**	**X**			
**08. Was the data analysis sufficiently rigorous?**	**X**			
**09. Is there a clear statement of findings?**	**X**			
Section C: Will the results help locally?				
**10. How valuable is the research?**	Broad discussion of own findings with other studies, limitations are reflected and conclusions are drawn.			

Karlsson et al., 2012 B				
Question	**Yes**	**Can't tell**	**No**	**Comments**
Section A: Are the results valid?				
**01. Was there a clear statement of the aims of the research?**	X			
**02. Is a qualitative methodology appropriate?**	X			
**03. Was the research design appropriate to address the aims of the research?**	X			
**04. Was the recruitment strategy appropriate to the aims of the research?**	X			
**05. Was the data collected in a way that addressed the research issue?**	X			
**06. Has the relationship between researcher and participants been adequately considered?**			X	No information provided.
Section B: What are the results?				
**07. Have ethical issues been taken into consideration?**	X			
**08. Was the data analysis sufficiently rigorous?**	X			
**09. Is there a clear statement of findings?**	X			
Section C: Will the results help locally?				
**10. How valuable is the research?**	The authors discuss their findings and their methodological approach separated from each other, thereby subsuming the limitations of their study in the discussion parts; conclusions are drawn.			

Kjeldsen et al., 2017				
Question	**Yes**	**Can't tell**	**No**	**Comments**
Section A: Are the results valid?				
**01. Was there a clear statement of the aims of the research?**	**X**			
**02. Is a qualitative methodology appropriate?**	**X**			
**03. Was the research design appropriate to address the aims of the research?**	**X**			
**04. Was the recruitment strategy appropriate to the aims of the research?**	**X**			
**05. Was the data collected in a way that addressed the research issue?**	**X**			
**06. Has the relationship between researcher and participants been adequately considered?**			**X**	No information provided.
Section B: What are the results?				
**07. Have ethical issues been taken into consideration?**	**X**			
**08. Was the data analysis sufficiently rigorous?**	**X**			
**09. Is there a clear statement of findings?**	**X**			
Section C: Will the results help locally?				
**10. How valuable is the research?**	Broad discussion of own results with other studies, limitations are reflected, implications and recommendations are formulated and conclusions are drawn.			

Laerkner et al., 2017				
Question	**Yes**	**Can't tell**	**No**	**Comments**
Section A: Are the results valid?				
**01. Was there a clear statement of the aims of the research?**	**X**			
**02. Is a qualitative methodology appropriate?**	**X**			
**03. Was the research design appropriate to address the aims of the research?**	**X**			
**04. Was the recruitment strategy appropriate to the aims of the research?**	**X**			
**05. Was the data collected in a way that addressed the research issue?**	**X**			Limitation: Initial interviews mainly consisted of yes/no questions.
**06. Has the relationship between researcher and participants been adequately considered?**		**X**		The field researcher and her experience are clearly stated, but no information on the relationship/ interaction with patients is provided.
Section B: What are the results?				
**07. Have ethical issues been taken into consideration?**	**X**			
**08. Was the data analysis sufficiently rigorous?**	**X**			
**09. Is there a clear statement of findings?**	**X**			
Section C: Will the results help locally?				
**10. How valuable is the research?**	Broad discussion of own findings with other studies, limitations are reflected and conclusions are drawn.			

**Lehmkuhl et al., 2023**				
Question	**Yes**	**Can't tell**	**No**	**Comments**
Section A: Are the results valid?				
**01. Was there a clear statement of the aims of the research?**	**X**			
**02. Is a qualitative methodology appropriate?**	**X**			
**03. Was the research design appropriate to address the aims of the research?**	**X**			
**04. Was the recruitment strategy appropriate to the aims of the research?**	**X**			
**05. Was the data collected in a way that addressed the research issue?**	**X**			
**06. Has the relationship between researcher and participants been adequately considered?**		**X**		Only implicit information through the description of methodological approach.
Section B: What are the results?				
**07. Have ethical issues been taken into consideration?**	**X**			
**08. Was the data analysis sufficiently rigorous?**	**X**			
**09. Is there a clear statement of findings?**	**X**			
Section C: Will the results help locally?				
**10. How valuable is the research?**	Broad discussion of own findings with other studies, limitations are reflected and conclusions are drawn.			

Schou et al., 2008				
Question	Yes	Can't tell	No	Comments
Section A: Are the results valid?				
**01. Was there a clear statement of the aims of the research?**	X			
**02. Is a qualitative methodology appropriate?**	X			
**03. Was the research design appropriate to address the aims of the research?**	X			
**04. Was the recruitment strategy appropriate to the aims of the research?**	X			
**05. Was the data collected in a way that addressed the research issue?**	X			
**06. Has the relationship between researcher and participants been adequately considered?**	X			
Section B: What are the results?				
**07. Have ethical issues been taken into consideration?**	X			
**08. Was the data analysis sufficiently rigorous?**	X			
**09. Is there a clear statement of findings?**	X			
Section C: Will the results help locally?				
**10. How valuable is the research?**	Broad discussion of own findings with other studies, limitations are reflected and conclusions are drawn.			

Tingsvik et al., 2018				
Question	**Yes**	**Can't tell**	**No**	**Comments**
Section A: Are the results valid?				
**01. Was there a clear statement of the aims of the research?**	**X**			
**02. Is a qualitative methodology appropriate?**	**X**			
**03. Was the research design appropriate to address the aims of the research?**	**X**			
**04. Was the recruitment strategy appropriate to the aims of the research?**	**X**			
**05. Was the data collected in a way that addressed the research issue?**	**X**			
**06. Has the relationship between researcher and participants been adequately considered?**	**X**			
Section B: What are the results?				
**07. Have ethical issues been taken into consideration?**	**X**			
**08. Was the data analysis sufficiently rigorous?**	**X**			
**09. Is there a clear statement of findings?**	**X**			
Section C: Will the results help locally?				
**10. How valuable is the research?**	Broad discussion of own findings with other studies, limitations are reflected and conclusions are drawn.			

Wang et al., 2009				
Question	**Yes**	**Can't tell**	**No**	**Comments**
Section A: Are the results valid?				
**01. Was there a clear statement of the aims of the research?**	**X**			
**02. Is a qualitative methodology appropriate?**	**X**			
**03. Was the research design appropriate to address the aims of the research?**	**X**			
**04. Was the recruitment strategy appropriate to the aims of the research?**	**X**			
**05. Was the data collected in a way that addressed the research issue?**	**X**			
**06. Has the relationship between researcher and participants been adequately considered?**	**X**			
Section B: What are the results?				
**07. Have ethical issues been taken into consideration?**	**X**			
**08. Was the data analysis sufficiently rigorous?**	**X**			
**09. Is there a clear statement of findings?**	**X**			
Section C: Will the results help locally?				
**10. How valuable is the research?**	Very broad discussion of own findings with other studies, in parts not that near to the research subject; limitations are in parts redundant and not worth mentioning, conclusion is short.			

Summary of all appraised studies

Study	Item 1	Item 2	Item 3	Item 4	Item 5	Item 6	Item 7	Item 8	Item 9	Value of the research (Item 10)
Aslani, 2017	+	+	+	+	+	-	+	+	+	The study highlights the psychological impact of mechanical ventilation and the sensibility for patients' needs. It discusses its results with other studies and recommends certain aspects to clinicians.
Ballard, 2006	+	+	+	+	+	-	+	+	+	Research explores a new field, that has not been investigated yet. Results are discussed with other studies, recommendations are made for clinicians.
Dale, 2020	+	+	+	+	+	-	+	+	+	Creative research combining interview methods and object elicitation (e.g. tube) to provide in-depth insights. Results are discussed with other studies, limitations are reflected and conclusions are drawn.
Donnelly, 2006	+	+	+	?	+	?	+	+	+	Methodological reflection, but no discussion of results with other studies; limitations also reflect methods only.
Engström, 2013	+	+	+	+	+	-	+	+	+	Rich discussion comparing own results with other studies, own limitations are reflected and conclusions are drawn.
Gilder, 2021	+	+	+	+	+	+	+	+	+	Broad discussion of own results with other studies, limitations are highlighted and conclusions are drawn.
Guttormson, 2015	+	+	?	?	-	-	+	+	+	Results are discussed with other studies, short information on limitations is provided, conclusions are short, but in line with study results.
Hajiabadi, 2017	+	+	+	+	+	+	+	+	+	Broad discussion of results with other studies, limitations are not sufficiently presented and lacking other potential biases; implications for practice are formulated.
Hajiabadi, 2018	+	+	+	+	+	+	+	+	+	Broad discussion of results with other studies, sufficient reflection of limitations, conclusions are drawn.
Holm, 2015	+	+	+	+	+	?	+	+	+	Broad discussion of findings with other studies, limitations are subsumed under methodological considerations, conclusions and implications are formulated.
Holm, 2017	+	+	+	+	+	?	+	+	+	Broad discussion of findings with other studies, limitations are reflected as well as the rigour of the study, implications and recommendations for practice are presented and conclusions are drawn.
Johnson, 2006	+	+	+	+	+	-	+	+	+	The focus of the discussion is not comprehensible in some places, e.g. the pathogenesis of delirium or confusion is discussed. No limitations are given, but conclusions can be found.
Karlsson, 2012 A	+	+	+	?	+	-	+	+	+	Broad discussion of own findings with other studies, limitations are reflected and conclusions are drawn.
Karlsson, 2012 B	+	+	+	+	+	-	+	+	+	The authors discuss their findings and their methodological approach separated from each other, thereby subsuming the limitations of their study in the discussion parts; conclusions are drawn.
Kjeldsen, 2017	+	+	+	+	+	-	+	+	+	Broad discussion of own results with other studies, limitations are reflected, implications and recommendations are formulated and conclusions are drawn.
Laerkner, 2017	+	+	+	+	+	?	+	+	+	Broad discussion of own findings with other studies, limitations are reflected and conclusions are drawn.
Lehmkuhl, 2023	+	+	+	+	+	?	+	+	+	Broad discussion of own findings with other studies, limitations are reflected and conclusions are drawn.

Schou, 2008	+	+	+	+	+	+	+	+	+	Broad discussion of own findings with other studies, limitations are reflected and conclusions are drawn.
Tingsvik, 2018	+	+	+	+	+	+	+	+	+	Broad discussion of own findings with other studies, limitations are reflected and conclusions are drawn.
Wang, 2009	+	+	+	+	+	+	+	+	+	Very broad discussion of own findings with other studies, in parts not that near to the research subject; limitations are in parts redundant and not worth mentioning, conclusion is short.
