# Supplementary material for: Being an observer of one’s own life—a meta-synthesis on the experience of mechanically ventilated patients in intensive care units
Source: Crit Care. 2025 Mar 8;29:105. doi: 10.1186/s13054-025-05326-6 (PMC11889880; doi:10.1186/s13054-025-05326-6)
Supplement: Supplementary file 3 — Additional file3 (PDF 214 KB) [file 13054_2025_5326_MOESM3_ESM.pdf]

Additional file 3: Excluded studies in fulltext screening

Meta-synthesis on the experience of ventilated ICU patients

Nr.	Excluded study	Reason
01	Alpers, L. M., Helseth, S., & Bergbom, I. (2012). Experiences of inner strength in critically ill patients--a hermeneutical approach. *Intensive & critical care nursing, 28*(3), 150–158. https://doi.org/10.1016/j.iccn.2011.10.004	Wrong scope: Experience of entire ICU stay, no differentiation between ventilated and spontaneous breathing times
02	Ashkenazy, S., Weissman, C., & DeKeyser Ganz, F. (2021). Perception of discomfort by mechanical ventilation patients in the Intensive Care Unit: A qualitative study. *Intensive & critical care nursing, 64*, 103016. https://doi.org/10.1016/j.iccn.2021.103016	Wrong scope: Study investigates the correlation of pain and discomfort
03	Berntzen, H., Bjørk, I. T., & Wøien, H. (2018). "Pain relieved, but still struggling"-Critically ill patients experiences of pain and other discomforts during analgosedation. *Journal of clinical nursing, 27*(1-2), e223–e234. https://doi.org/10.1111/jocn.13920	Wrong scope: Study investigates correlation of pain and discomfort, but no relation to MV; experience after MV is also included
04	Chen, C. J., Lin, C. J., Tzeng, Y. L., & Hsu, L. N. (2009). Successful mechanical ventilation weaning experiences at respiratory care centers. *The journal of nursing research : JNR, 17*(2), 93–101. https://doi.org/10.1097/JNR.0b013e3181a6a601	Wrong setting: Respiratory care unit in a medical center including patients with at least 3 weeks of ventilation before admission/inclusion
05	Claesson, A., Mattson, H., & Idvall, E. (2005). Experiences expressed by artificially ventilated patients. *Journal of clinical nursing, 14*(1), 116–117. https://doi.org/10.1111/j.1365-2702.2004.00948.x	Poor description of qualitative study results
06	Fink, R. M., Makic, M. B., Poteet, A. W., & Oman, K. S. (2015). The Ventilated Patient's Experience. *Dimensions of critical care nursing : DCCN, 34*(5), 301–308. https://doi.org/10.1097/DCC.0000000000000128	Poor description of qualitative study results
07	Flinterud, S. I., & Andershed, B. (2015). Transitions in the communication experiences of tracheostomised patients in intensive care: a qualitative descriptive study. *Journal of clinical nursing, 24*(15-16), 2295–2304. https://doi.org/10.1111/jocn.12826	Wrong study design: Interviews conducted until 18 months after MV in ICU
08	Forbes M. O. (2007). Prolonged ventilator dependence: perspective of the chronic obstructive pulmonary disease patient. *Clinical nursing research, 16*(3), 231–250. https://doi.org/10.1177/1054773807302781	Wrong setting: Long-term ventilated patients included

09	Fowler S. B. (1997). Impaired verbal communication during short-term oral intubation. *Nursing diagnosis : ND : the official journal of the North American Nursing Diagnosis Association, 8*(3), 93–98. https://doi.org/10.1111/j.1744-618x.1997.tb00317.x	Poor description of qualitative study results
10	Grap, M. J., Blecha, T., & Munro, C. (2002). A description of patients' report of endotracheal tube discomfort. *Intensive & critical care nursing, 18*(4), 244–249. https://doi.org/10.1016/s0964339702000654	Poor description of qualitative study results
11	Hafsteindóttir T. B. (1996). Patient's experiences of communication during the respirator treatment period. *Intensive & critical care nursing, 12*(5), 261–271. https://doi.org/10.1016/s0964-3397(96)80693-8	Wrong study design: Unclear time of data collection
12	Haugdahl, H. S., Dahlberg, H., Klepstad, P., & Storli, S. L. (2017). The breath of life. Patients' experiences of breathing during and after mechanical ventilation. *Intensive & critical care nursing, 40*, 85–93. https://doi.org/10.1016/j.iccn.2017.01.007	Wrong population: Non-intubated patients included
13	Jablonski, R. S. (1994). The Experience of Being Mechanically Ventilated. *Qualitative Health Research, 4*(2), 186-207. https://doi.org/10.1177/104973239400400204	Wrong study design: Interviews conducted until 108 months after MV in ICU
14	Johansson, L., & Fjellman-Wiklund, A. (2005). Ventilated patients' experiences of body awareness at an intensive care unit. *Advances in Physiotherapy, 7*(4), 154-161. https://doi.org/10.1080/14038190500205808	Wrong study design: Interviews conducted until 6 years after MV in ICU
15	Johnson P. (2004). Reclaiming the everyday world: how long-term ventilated patients in critical care seek to gain aspects of power and control over their environment. *Intensive & critical care nursing, 20*(4), 190–199. https://doi.org/10.1016/j.iccn.2004.05.001	Wrong study design: Unclear time of data collection
16	Jordan, P. J., van Rooyen, D., & Strümpher, J. (2002). The lived experience of patients on mechanical ventilation. *Health SA Gesondheit, 7*(4), 24-37. https://doi.org/10.4102/hsag.v7i4.101	Poor description of qualitative study results
17	Karlsson, V., & Forsberg, A. (2008). Health is yearning--experiences of being conscious during ventilator treatment in a critical care unit. *Intensive & critical care nursing, 24*(1), 41–50. https://doi.org/10.1016/j.iccn.2007.06.004	Wrong study design: Unclear time of data collection
18	Khalafi, A., Elahi, N., & Ahmadi, F. (2016). Continuous care and patients' basic needs during weaning from mechanical ventilation: A qualitative study. *Intensive & critical care nursing, 37*, 37–45. https://doi.org/10.1016/j.iccn.2016.05.005	Wrong population: Staff perspective
19	Logan, J., & Jenny, J. (1997). Qualitative analysis of patients' work during mechanical ventilation and weaning. *Heart & lung : the journal of critical care, 26*(2), 140–147. https://doi.org/10.1016/s0147-9563(97)90074-1	Poor description of qualitative study results; no transparent methodological approach

20	Ma, P., Liu, J., Xi, X., Du, B., Yuan, X., Lin, H., Wang, Y., Su, J., & Zeng, L. (2010). Practice of sedation and the perception of discomfort during mechanical ventilation in Chinese intensive care units. *Journal of critical care, 25*(3), 451–457. https://doi.org/10.1016/j.jcrc.2009.11.006	Wrong study design: Quantitative analysis only
21	Marasinghe, M. S. P., Fonseka , W. I. T., Wanishri , P. C., Nissanka , N. K. S. M., & De Silva, B. S. S. (2015). An Exploration of Patients ' Experiences of Mechanical Ventilation. OUSL Journal, 9 , 83 96. https://doi.org/10.4038/ouslj.v9i0.7328	Poor description of qualitative results
22	Pakmehr, M., Rahnama, M., Firouzkouhi, M. R., Abollahimohammad, A. (2017). Lived Experience of Intubated Patients: A Phenomenological Study. *Indian Journal of Public Health Research & Development, 8*(1). 286-291. https://doi.org/10.5958/0976-5506.2017.00059.6	Wrong study design: Unclear time of data collection
23	Patak, L., Gawlinski, A., Fung, N. I., Doering, L., & Berg, J. (2004). Patients' reports of health care practitioner interventions that are related to communication during mechanical ventilation. *Heart & lung : the journal of critical care, 33*(5), 308–320. https://doi.org/10.1016/j.hrtlng.2004.02.002	Poor description of qualitative results
24	Perez, D., Murphy, G., Wilkes, L., & Peters, K. (2022). Being tied down-The experience of being physically restrained while mechanically ventilated in ICU. *Journal of advanced nursing, 78*(11), 3760–3771. https://doi.org/10.1111/jan.15354	Wrong study design: Interviewed patients' mechanical ventilation in ICU took place 'years to decades ago'
25	Prime, D., Arkless, P., Fine, J., Winter, S., Wakefield, D. B., & Scatena, R. (2016). Patient experiences during awake mechanical ventilation. *Journal of community hospital internal medicine perspectives, 6*(1), 30426. https://doi.org/10.3402/jchimp.v6.30426	Wrong study design: Quantitative analysis only
26	Roberts, M., Bortolotto, S. J., Weyant, R. A., Jock, L., LaLonde, T., & Henderson, A. (2019). The Experience of Acute Mechanical Ventilation From the Patient's Perspective. *Dimensions of critical care nursing : DCCN, 38*(4), 201–212. https://doi.org/10.1097/DCC.0000000000000361	Poor description of qualitative results
27	Rotondi, A. J., Chelluri, L., Sirio, C., Mendelsohn, A., Schulz, R., Belle, S., Im, K., Donahoe, M., & Pinsky, M. R. (2002). Patients' recollections of stressful experiences while receiving prolonged mechanical ventilation in an intensive care unit. *Critical care medicine, 30*(4), 746–752. https://doi.org/10.1097/00003246-200204000-00004	Wrong study design: Quantitative analysis only
28	Samuelson K. A. (2011). Unpleasant and pleasant memories of intensive care in adult mechanically ventilated patients--findings from 250 interviews. *Intensive & critical care nursing, 27*(2), 76–84. https://doi.org/10.1016/j.iccn.2011.01.003	Poor description of qualitative results
29	Takashima, N., Yosihno, Y., & Sakaki, K. (2019). Quantitative and qualitative investigation of the stress experiences of intensive care unit patients mechanically ventilated for more than 12 hr. *Japan journal of nursing science : JJNS, 16*(4), 468–480. https://doi.org/10.1111/jjns.12253	Wrong study design: Missing distinction between qualitative and quantitative parts

30	Tate, J. A., Devito Dabbs, A., Hoffman, L. A., Milbrandt, E., & Happ, M. B. (2012). Anxiety and agitation in mechanically ventilated patients. *Qualitative health research, 22*(2), 157–173. https://doi.org/10.1177/1049732311421616	Wrong study design: Also reports from health care professionals included and no distinction between patients' and HCPs' reports is conducted.
31	Tembo, A. C., Higgins, I., & Parker, V. (2015). The experience of communication difficulties in critically ill patients in and beyond intensive care: Findings from a larger phenomenological study. *Intensive & critical care nursing, 31*(3), 171–178. https://doi.org/10.1016/j.iccn.2014.10.004	Wrong study design: Interviews conducted until 11 months after MV in ICU
32	Wojnicki-Johansson G. (2001). Communication between nurse and patient during ventilator treatment: patient reports and RN evaluations. *Intensive & critical care nursing, 17*(1), 29–39. https://doi.org/10.1054/iccn.2000.1547	Wrong study design: Missing distinction between qualitative and quantitative parts
33	Wunderlich, R. J., Perry, A., Lavin, M. A., & Katz, B. (1999). Patients' perceptions of uncertainty and stress during weaning from mechanical ventilation. *Dimensions of critical care nursing : DCCN, 18*(1), 8–12.	Poor description of qualitative results
