# Supplementary material for: Being an observer of one’s own life—a meta-synthesis on the experience of mechanically ventilated patients in intensive care units
Source: Crit Care. 2025 Mar 8;29:105. doi: 10.1186/s13054-025-05326-6 (PMC11889880; doi:10.1186/s13054-025-05326-6)
Supplement: Supplementary file 4 — Additional file4 (PDF 133 KB) [file 13054_2025_5326_MOESM4_ESM.pdf]

Additional file 4: Meta-summary of studies

Main concepts	Nr.	Classified findings	Study sources	Frequent effect sizes	Codings
Being an observer of one's own life	#1	*Patients are* noticing the changes of their situation	13/20	65 %	38
	#2	*Patients are* not being able to	18/20	90 %	124
	#3	*Patients are* being at the mercy of the situation	15/20	75 %	44
	#4	*Patients are* living through an existential crisis	17/20	85 %	88
	#5	*Patients are* anticipating future	17/20	85 %	55
Yearning for a stable picture of reality	#6	*Patients are* becoming aware of their own situation	20/20	100 %	6
	#6.1	*Patients are* realizing the misinterpretation of the situation	18/20	90 %	139
	#6.2	*Patients are* being aware of their own situation	20/20	100 %	703
	#6.3	*Patients are* being aware of their own improvements	11/20	55 %	50
	#7	*Patients are* differentiating their own emotions	20/20	100 %	271
	#8	*Patients are* perceiving various bodily sensations	20/20	100 %	254
	#9	*Patients are* trying to make sense	14/20	70 %	46
Developing various situation-specific needs	#10	*Patients are* having basic needs	15/20	75 %	72
	#11	*Patients are* striving for an understanding of their own health status	13/20	65 %	36
	#12	*Patients are* longing for personal integrity	15/20	75 %	68
	#13	*Patients are* longing for social interaction	10/20	50 %	64
Finding ways to deal with the situation	#14	*Patients are* having to cope internally alone	19/20	95 %	3
	#14.1	*Patients are* being confronted with their own perception	19/20	95 %	129
	#14.2	*Patients are* coping with their current situation	15/20	75 %	33
	#15	*Patients are* trying to satisfy their needs themselves	20/20	100 %	206
	#16	*Patients are* getting their needs fulfilled by others	14/20	70 %	52
	#17	*Patients are* evaluating strategies depending on the situation	20/20	100 %	350
	#18	*Patients are* drawing consequences from the situation	20/20	100 %	227
